# Comparison of Immunogenicity and Safety of Four Doses and Four Double Doses vs. Standard Doses of Hepatitis B Vaccination in HIV-Infected Adults: A Randomized, Controlled Trial 

**DOI:** 10.1371/journal.pone.0080409

**Published:** 2013-11-12

**Authors:** Kanokporn Chaiklang, Jiraprapa Wipasa, Romanee Chaiwarith, Jutarat Praparattanapan, Khuanchai Supparatpinyo

**Affiliations:** 1 Department of Medicine, Faculty of Medicine, Chiang Mai University, Chiang Mai, Thailand; 2 Research Institutes for Health Sciences, Chiang Mai University, Chiang Mai, Thailand; University of New South Wales, Australia

## Abstract

**Background:**

HBV vaccination is recommended in HIV-infected adults with CD4+ cell count >200/mm^3^ although the efficacy is only 33.3% -65%. We conducted a randomized, controlled trial to evaluate the efficacy and safety of three regimens of HBV vaccination at Chiang Mai University Hospital, Thailand.

**Methods:**

From February 4, 2011 to May 4, 2012, 132 HIV-infected adults with CD4+ cell counts >200 cells/mm^3^, undetectable plasma HIV-1 RNA, and negative for all HBV markers were randomly assigned to receive one of three recombinant vaccine (Hepavax-Gene^®^ Berna, Korea) regimens: 20 μg IM at months 0, 1, and 6 (Standard doses group, n=44), 20 μg IM at months 0, 1, 2, 6 (four doses group, n=44), or 40 μg IM at months 0, 1, 2, and 6 (four double doses group, n=44). The primary outcomes were to compare the immunogenicity and safety between the four-doses groups with the Standard doses group.

**Results:**

At months 7 and 12, the percentages of responders (anti-HBs ≥10 mIU/mL) were 88.6% and 70.4% in the Standard doses group, 93.2% and 86.4% in the four doses group, (*P*=0.713 and 0.119), and 95.4% and 88.6% in the four double doses group, (*P*=0.434 and 0.062), respectively. Factors associated with a high titer level (anti-HBs ≥100 mIU/mL) were vaccination schedule and younger age. The most common adverse event was pain at the injection site (42.4%); this was significantly more frequent in the four double doses group compared to the Standard doses group. No serious adverse events were observed.

**Conclusions:**

In Northern Thailand, the standard three-doses HBV vaccination in HIV-infected adults with CD4+ cell counts >200 cells/mm^3^ and undetectable plasma HIV-1 RNA is highly effective. Although regimens of four injections of either standard or double doses could not significantly increase the response rate, these regimens may induce higher levels of antibody to the virus.

Trial registration information: ClinicalTrials.gov; NCT1289106; http://clinicaltrials.gov/ct2/show/NCT01289106

## Introduction

The human immunodeficiency virus (HIV) and hepatitis B virus (HBV) share similar risk factors and routes of transmission, resulting in a high prevalence of co-infection, particularly in resource limited countries [1,2]. The progression of chronic HBV to cirrhosis, end-stage liver disease, and hepatocellular carcinoma is more rapid in HIV-infected individuals than in those with chronic HBV alone [3,4]. Vaccination against HBV is strongly recommend in HIV infected persons who have CD4+ cell count ≥200 cells/mm^3^ [5,6], although the efficacy of hepatitis B vaccine in HIV-infected adults is low, i.e., from 33.3% to 65% [7–10] compared with more than 90% in HIV-negative healthy adults [11,12]. HBV vaccination responses vary directly on CD4+ counts [10,13–15]. The HIV-HBV International Panel recommends that vaccination should not be attempted in participants with CD4+ cell counts <200 cells/mm^3^ as protective immune response is poor in these participants. Combination antiretroviral therapy (cART) should be started first and HBV immunization should be delayed until the CD4+ cell count has increased to above 200 cells/mm^3^ [16]. Several approaches have been investigated to improve HBV vaccine responses, e.g., higher dose [15], increased frequency [9,17], increased dose and frequency [10,14], or intradermal injection [10]. However, there was a heterogeneity of populations and a mix in the responses to HBV vaccination among those studies, as well as the technical issue of multiple intradermal injections of vaccine. Therefore, up to date there remains no conclusive evidence to make firm recommendations regarding optimal doses, routes, and frequency of HBV vaccination in HIV-infected individuals. In Thailand, the current clinical practice guidelines, endorsed by the Royal College of Physicians of Thailand, recommends giving three intramuscular injections of 20 µg of recombinant HBV vaccine at months 0, 1, and 6, to all adults regardless of underlying diseases and immune status [18].

The primary objectives of this study were to evaluate the efficacy and safety of the HBV vaccination regimens using either four standard doses or four double doses compared with the current standard regimen of three doses in HIV-infected adults in northern Thailand. The secondary objectives were to evaluate the geometric means of anti-HBs titers and the percentages of responders with high level of immune response among those three HBV vaccination regimens.

## Methods

The protocol for this trial including English translation and supporting CONSORT checklist are available as supporting information; see Protocol S1, Protocol S2, and Checklist S1.

### Study design and participants

A randomized, open-label, controlled trial, 1:1:1 allocation ratio was conducted at Chiang Mai University Hospital, Chiang Mai, Thailand between February 4, 2011 and May 4, 2012. Participants were eligible to participate if they were adults with HIV-1 infection, ≥18 years old, had a CD4+ cell count >200 cells/mm^3^, undetectable plasma HIV-1 RNA, were negative for hepatitis B surface antigen (HBsAg), antibody to hepatitis B surface antigen (anti-HBs), and antibody to hepatitis B core antigen (anti-HBc), had no history of previous vaccine, were negative for antibody to hepatitis C virus (anti-HCV), had no active opportunistic infections (at the time of screening), and were willing to sign an informed consent and able to return for follow-up.

The exclusion criteria included pregnancy or lactation, history of hypersensitivity to any component of the vaccine, active malignancy receiving chemotherapy or radiation, other immunocompromised conditions besides HIV (e.g., solid organ transplant), received immunosuppressive (e.g., corticosteroid ≥ 0·5 mg/kg/day) or immunomodulating treatment in the last six months before screening visit, had renal insufficiency (creatinine clearance <30 mL/min), decompensated cirrhosis (Child-Pugh class C) [19].

Written informed consent was obtained before enrollment. The study was approved by the Research Ethics Committee 1, Faculty of Medicine, Chiang Mai University and the Human Experimentation Committee, Research Institute for Health Sciences, Chiang Mai University. Awaiting the official approval letters from both ethic committees led to a brief delay in registration to the ClinicalTrials.gov (NCT1289106, 1 February 2011); however, subject enrollment was started right after the trial approval and registration.

### Data collection

Baseline demographic data at the time of screening were recorded including age, sex, weight, height, HIV exposure category, history of smoking, history of alcohol drinking and underlying disease. Medical data included time elapsed since HIV diagnosis, duration of cART, current cART regimen and history of HIV drug resistance. Baseline laboratory data included CD4+ cell count, plasma HIV-1 RNA, serum creatinine, HBsAg, anti-HBs, anti-HBc and anti-HCV. 

### Randomization and masking

 JP prepared a list for randomization to one of the three arms in a 1:1:1 ratio with a block size of 6 using sequentially numbered containers. Randomization was not stratified by any characteristics. Participants, investigators, laboratory technicians, and statistician were not masked to the treatment allocation.

### Interventions

Participants were randomized (1:1:1) by block of six into 3 groups: 1) the “Standard doses group” receiving three intramuscular injections of 20 μg of recombinant HBV vaccine (Hepavax-Gene^®^ Berna, Korea) at months 0, 1, and 6; 2) the “Four doses group” receiving four intramuscular doses of 20 μg of the same vaccine at months 0, 1, 2, and 6; or 3) the “Four double doses group” receiving four intramuscular double doses (40 μg) at months 0, 1, 2, and 6. The vaccine was injected into the deltoid muscle on patient preferential arm except in the Four double doses group which the vaccines were injected on both arms. The window period of vaccination was ±7 days. Blood samples were collected prior to each vaccination at months 0, 1, 2 (only for the Four doses and Four double doses group), 6, 7, and 12. The window period for anti-HBs titer at month 7 was ±14 days. The window period of anti-HBs titer at month 12 was ±30 days.

### Safety Assessment

Participants received a diary for self-record of adverse events after vaccinations. The diary contained checklists of occurrence and severity of local reactions at the injection site (redness, swelling, and pain), systemic reactions (fever, headache and fatigue), other adverse events during the ten days after vaccination, and an open field for any adverse event during the rest of period before the next vaccination.

### Laboratory Assays

Quantification of anti-HBs titers were performed on collected sera at the Research Institute for Health Sciences laboratory, Chiang Mai University, using a standardized assay (Microparticle enzyme immunoassay; AXSYM, Abbott, USA; sensitivity 100%; specificity 100%). Samples were tested by technical staff blinded to vaccine group allocation. Samples with titers of more than the upper quantification range of the assay were retested after dilution. HBs Ag, anti-HBc, and anti-HCV were determined by qualitative chemiluminescent microparticle immunoassay (CIMA; Architect^®^, Abbott, USA). The CD4+ cell counts were determined by a fluorescence-activated cell analyzer (FACScan^®^ System, USA). The plasma HIV-1 RNA was measured by COBAS AMPLICOR HIV-1 Monitor Test (version 1.5, Roche, Switzerland), with the lower limit of detection of 50 copies/ml. 

### Statistical Analysis

The primary efficacy endpoint was the percentage of responders (participants with anti-HBs titers ≥10 mIU/mL, levels presumptive for seroprotection) at one month after the last dose of vaccination (month 7). Secondary endpoints included the percentage of participants with seroprotective anti-HBs titers at months 1, 2 (for the Four doses and Four double doses groups), 6, and 12; the percentage of high-titer responders (participants with anti HBs titers ≥100 mIU/ml) at months 7 and 12; and the geometric means of anti-HBs titers at months 1, 2 (for the four doses regimens), 6, 7 and 12. The frequency and intensity of local and systemic adverse events were analyzed as safety endpoints. The intention-to-treat analyses were performed at the end of the study.

We tested whether the immunogenicity of either of two alternative vaccination regimens (Four doses and Four double doses groups) was higher than that of the Standard doses group. With the estimated seroconversion rates of 50% among the Standard doses group and 80% among the other two groups, the number of participants required in each group was 41 to detect such differences with a power of 80% and the level of significance of 5% (2-sided). To account for 5% loss to follow-up, a total of 44 participants in each group were recruited to the study.

Comparison of baseline characteristics between groups was performed using Chi-square test or Fisher’s exact test for categorical data and Student’s t-test or Mann-Whitney *U* test for continuous data. Proportions of participants with seroprotection, high-level responders, or adverse events between groups were compared using Chi-square test. Factors associated with achieving seroprotective antibody and high-titer antibody were tested in univariate models that included age, gender, mode of HIV transmission, underlying diseases, duration of HIV diagnosis, cART regimen and duration of therapy, history of HIV drug resistance, alcohol consumption, smoking, body mass index, CD4+ cell count, and creatinine clearance. Factors with the P-value <0.10 from univariate analysis were then tested in a multivariate logistic regression model. All statistical analyses were performed using Stata statistical software version 10.0 (Stata Statistical Software: Release 10.0, Stata Corporation, College Station, TX, 2007). A two-sided test was used to indicate statistical significance at a P value of <0.05.

## Results

### Study participants

**Figure 1:**
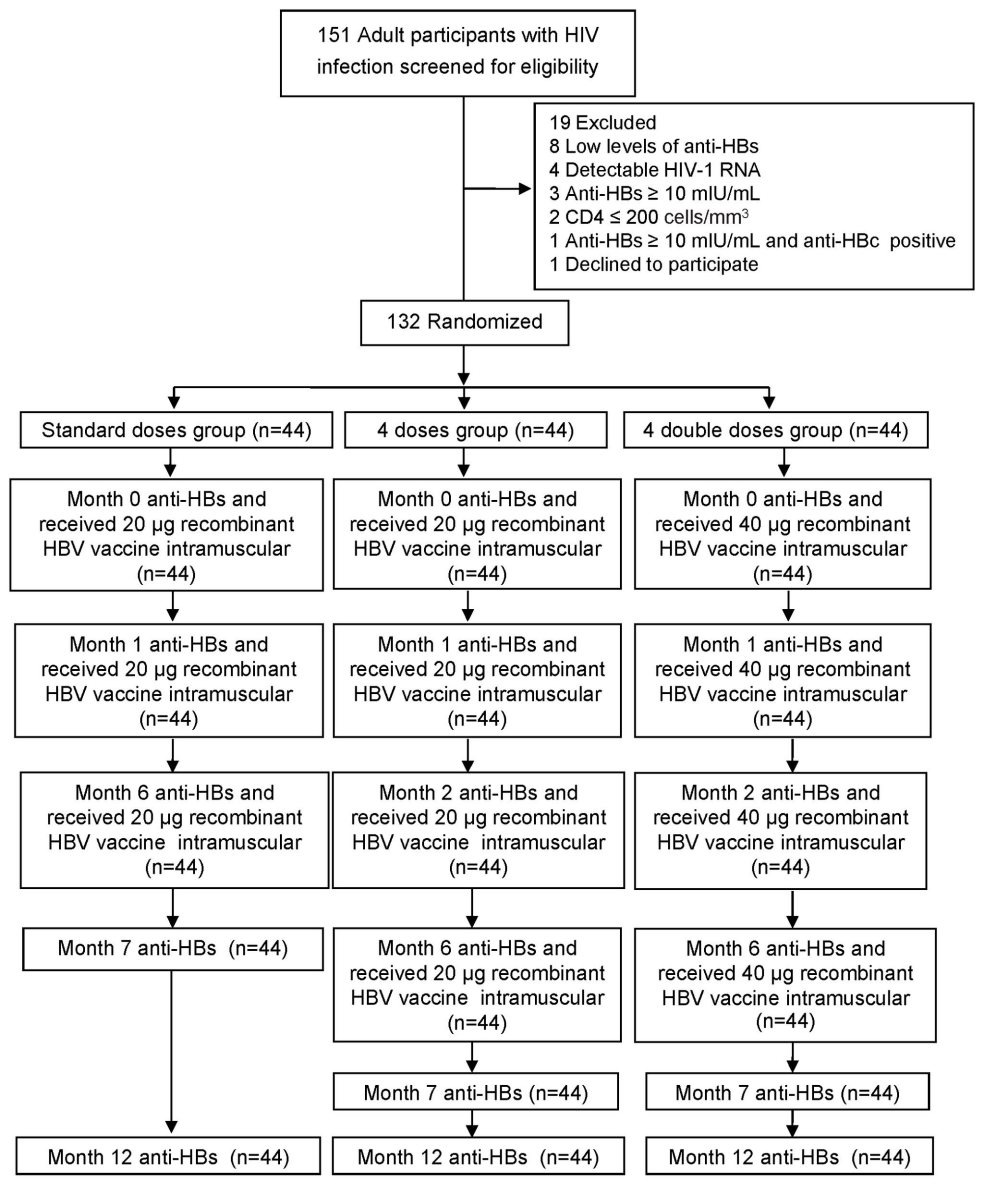
Consort diagram of participants.

Between February 4, 2011 and May 4, 2012, a total of 151 HIV-infected adults were screened for eligibility; 19 (12.6%) were excluded due to the presence of low level (<10 mIU/mL) of anti-HBs (8); plasma HIV-1 RNA >50 copies/mL (4); anti-HBs ≥10 mIU/mL (3); CD4+ cell count ≤200 cells/mm^3^ (2); anti-HBs ≥10 mIU/mL and positive for anti-HBc (1); and decline to participate (1). One hundred and thirty-two HIV-infected adults were randomized (44 each) to the Standard doses group, Four doses group, and Four double doses group (Figure 1). All 132 randomized participants received assigned vaccination and completed follow-up visits.

Demographic and clinical characteristics of participants by vaccination regimen were shown in Table 1. All participants completed the vaccination schedules and obtained anti-HBs titers at month 7 and month 12.

**Table 1:** Baseline demographics and clinical characteristics of participants by vaccination regimen.

**Characteristics**	**Standard doses (n=44)**	**4 doses (n=44**)	**P-value^¶^**	**4 double doses (n=44**)	**P-value^†^**
Female	36 (81.8%)	35 (79.6%)	1.000	25 (56.8%)	0.020
Age (years)	41.0 ± 6.3	42.2 ± 7.6	0.385	41.0 ± 6.2	0.631
Body mass index (kg/m^2^)	21.6 (20.4, 23.1)	21.1 (19.7, 23.4)	0.713	21.6 (19.3, 24.7)	0.438
Creatinine clearance (mL/min)	81.9 ± 22.5	83.1 ± 22.7	0.887	87.9 ± 21.7	0.223
CD4+ cell count	400 (314, 558)	544 (416, 731)	0.001	544 (410, 642)	0.004
CD4+ cell count by category					
CD4+ 201-350 cell/mm^3^	14 (31.8%)	5 (11.4)	0.036	5 (11.4)	0.036
CD4+ >350 cell/mm^3^	30 (68.2%)	39 (88.6)		39 (88.6)	
Nadir CD4+ cell count	70 (31, 143)	70 (33, 179)	0.576	90 (40, 173)	0.504
Time elapsed since HIV diagnosis (months)	134 (79, 182)	98 (83, 164)	0.435	120 (92, 170)	0.967
Current cART			1.000		1.000
NNRTI based	39 (88.6%)	39 (88.6%)		40 (90.9%)	
PI based	4 (9.1%)	4 (9.1%)		4 (9.1%)	
Others	1 (2.3%)	1 (2.3%)		0 (0%)	
Duration of cART (months)	80 (47, 90)	86 (64, 99)	0.187	92 (75, 110)	0.018
Duration of suppressed plasma HIV-1 RNA (months)	37.3 (27.2, 71.3)	73.2 (32.8, 76.8)	0.017	72.2 (34.2, 77.0)	0.017
History of drug resistance	3 (6.8%)	4 (9.1%)	1.000	2 (4.5%)	1.000
HIV exposure category			1.000		1.000
Heterosexual	42 (95.4%)	42 (95.4%)		43 (97.7%)	
Homosexual	1 (2.3%)	0 (0%)		1 (2.3%)	
IVDU	0 (0%)	1 (2.3%)		0 (0%)	
Blood transfusion	1 (2.3%)	0 (0%)		0 (0%)	
Unknown	0 (0%)	1 (2.3%)		0 (0%)	
Alcohol use			0.459		0.100
No	38 (86.3%)	34 (77.3%)		31 (70.5%)	
Social drinking	4 (9.1%)	8 (18.2%)		11 (25.0%)	
Regular drinking^a^	1 (2.3%)	2 (4.5%)		2 (4.5%)	
Heavy drinking^b^	1 (2.3%)	0 (0%)		0 (0%)	
Active smoking	2 (4.5%)	4 (9.1%)	0.676	4 (9.1%)	0.676
Underlying diseases					
Diabetes mellitus and IFG^c^	3 (6.8%)	5 (11.4%)	0.713	8 (18.1%)	0.196
Hypertension	3 (6.8%)	8 (18.2%)	0.196	10 (22.7%)	0.068
Dyslipidemia	7 (15.9%)	6 (13.6%)	1.000	7 (15.9%)	1.000
Others	5 (11.4%)	8 (18.2%)	0.549	8 (18.2%)	0.549

Data presented in number (%), means±SD, or median (IQR)

Abbreviation: cART, combination antiretroviral therapy; NNRTI, non-nucloside/nucleotide reverse transcriptase inhibitor; PI, protease inhibitor; IFG, impaired fasting glucose

^a^Defined as no more than 1 drink per day for women and no more than 2 drinks per day for men

^b^Defined as consuming an average of more than 1 drink per day for women and consuming an average of more than 2 drinks per day for men

^c^Defined as fasting plasma glucose from 5.6 mmol/L (100 mg/dL) to 6.9 mmol/L (125 mg/dL)

¶ Compare between the standard doses group and the 4 doses group

† Compare between the standard doses group and the 4 double doses group

### Immunogenicity

**Figure 2:**
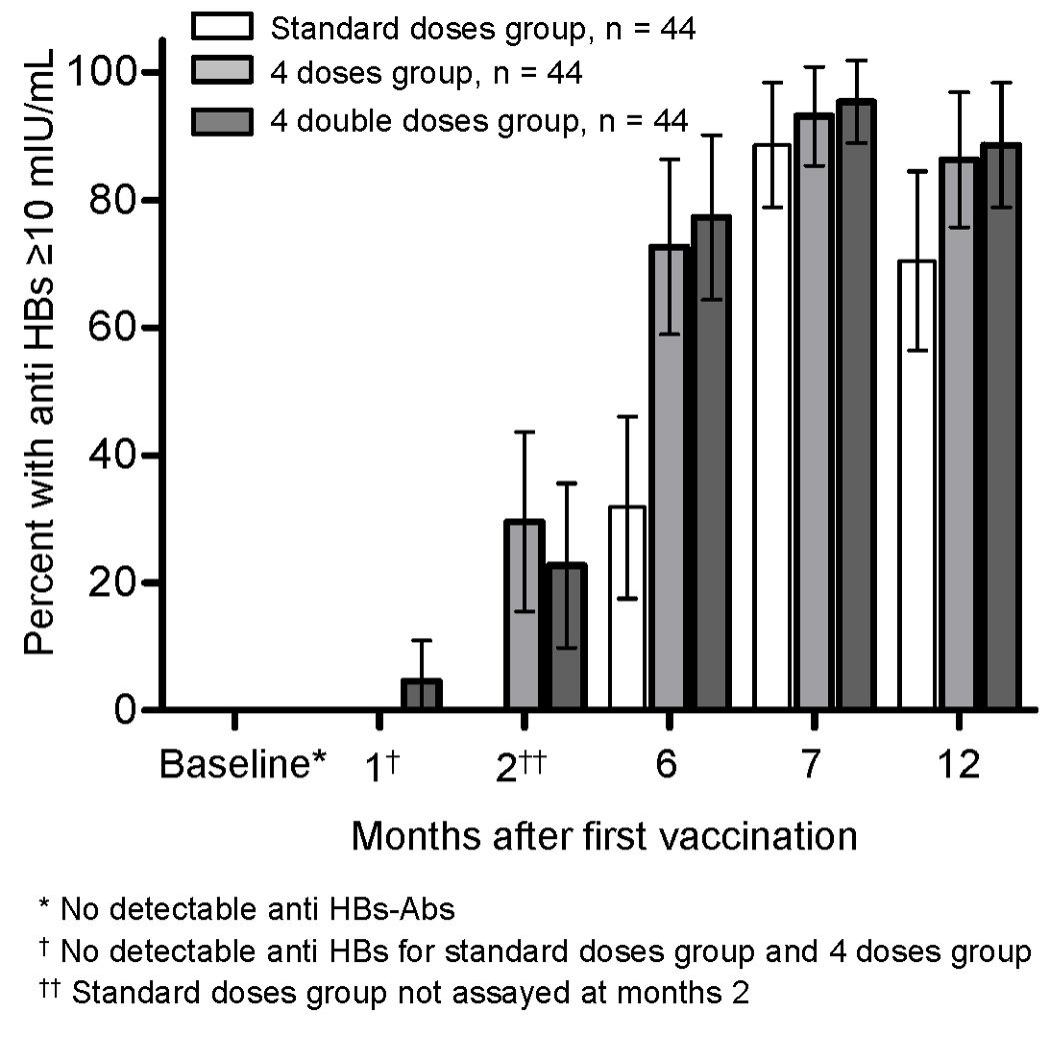
Percentages of responders (anti-HBs ≥ 10 mIU/ml) to hepatitis B vaccine by vaccination regimen.

At month 7 and 12, the percentages of responders (participants with anti-HBs titers ≥10 mIU/mL) were 88.6% (95% CI, 79.2%-98.0%) and 70.4% (57.0%-83.9%) in the Standard doses group; 93.2% (85.7%-100.6%) and 86.4% (76.2%-96.5%) in the Four doses group (*P*=0.713, 0.119 vs. the Standard group); and 95.4% (89.3%-101.6%) and 88.6% (79.2%-98.0%) in the Four double doses group (*P*=0.434, 0.062 vs. the Standard group), respectively (Figure 2). Differences of response rates at month 7 and 12 were 4.5% (-7.4%-16.5%) and 15.9% (-1.0%-32.8%) between the Four doses and the Standard doses groups; and 6.8% (-4.4%-18.0%) and 18.2% (1.8%-34.6%) between the Four double doses and the Standard doses groups, respectively.

**Figure 3:**
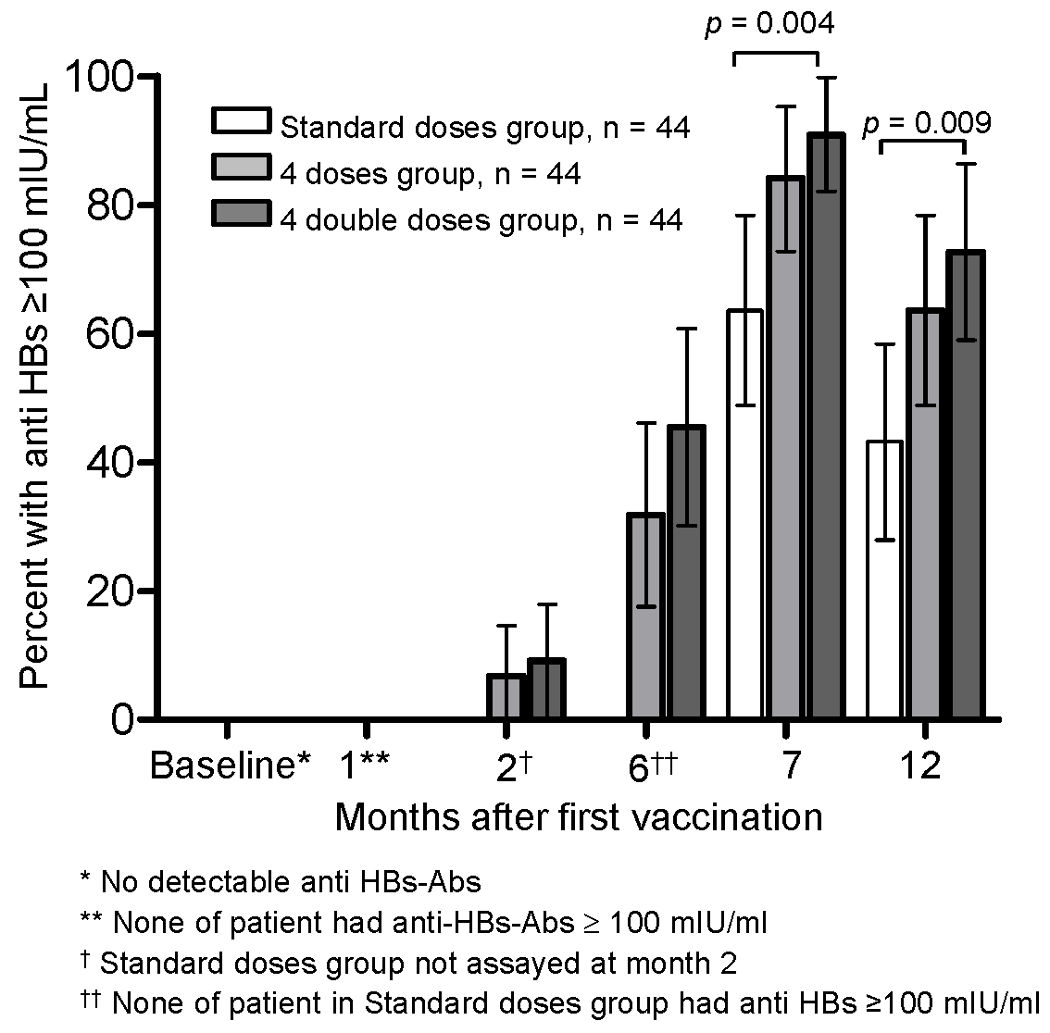
Percentages of responders (anti-HBs ≥ 100 mIU/ml) to hepatitis B vaccine by vaccination regimen.

At month 7 and 12, the percentages of high-titer responders (anti-HBs ≥100 mIU/mL) were 63.6% (95% CI, 49.4%-77.8%) and 43.2% (28.5%-57.8%) in the Standard doses group; 84.1% (73.3%-94.9%) and 63.6% (49.4%-77.9%) in the Four doses group (*P*=0.051 and 0.087 vs. the Standard doses group); and 90.9% (82.4%-99.4%) and 72.7% (59.6%-85.9%) in the Four double doses group (*P*=0.004 and 0.009 vs. the Standard doses group), respectively (Figure 3). 

**Figure 4:**
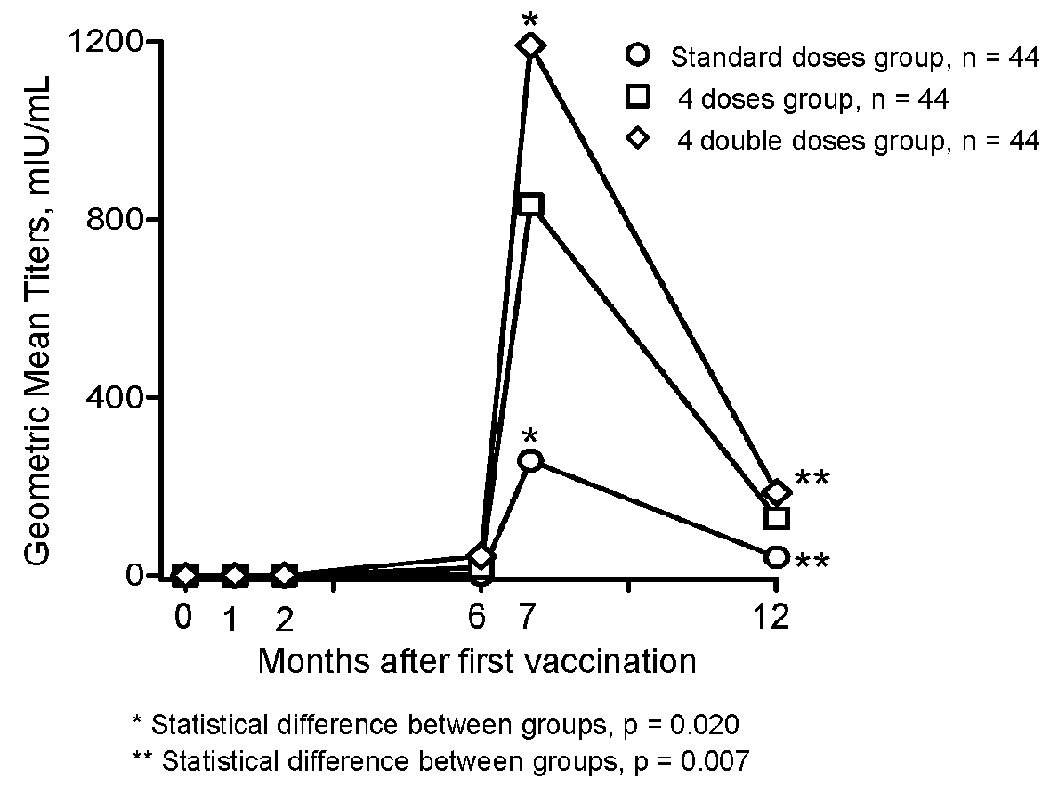
Geometric mean titers of anti-HBs antibody by vaccination regimen.

At month 7 and 12, the geometric means of anti-HBs titer were 257.6 mIU/mL (95% CI, 92.2-719.3) and 41.0 mIU/mL (16.8-100.1) in the Standard doses group; 833.9 mIU/mL (371.0-1874.5) and 128.9 mIU/mL (57.7-287.9) in the Four doses group (*P*=0.076 and 0.051 vs. the Standard doses group); and 1191.1 mIU/mL (576.2-2462.5) and 186.4 mIU/mL (103.0-337.2) in the Four double doses group (*P*=0.020 and 0.007 vs. the Standard doses group), respectively (Figure 4).

Factors associated with achieving a protective antibody level (anti-HBs ≥10 mIU/mL) was elapsed time from HIV diagnosis to antiretroviral therapy < 3 months. (Table 2). Multivariate analysis showed factors associated with a high titer level (anti-HBs ≥ 100 mIU/mL) were vaccination schedule (OR for the Four doses group vs. the Standard group = 3.71, 95% CI 1.27-10.85, OR for the Four double doses group vs. the Standard group = 6.19, 95% CI 1.81-21.21), and younger age (For every 5 years younger the odds of achieving high titer level increase 53%, 95% CI 8%-116%) (Table 3).

**Table 2:** Predictive factors from univariate analyses for responder (anti HBs ≥10 mIU/ml) at month 7.

**Factors**	**Responder† (n=122)**	**Non-responder (n=10)**	**Unadjusted *P*-value**
Vaccination schedule			0.603
Standard dose	39 (32.0%)	5 (50%)	
Four single dose	41 (33.6%)	3 (30%)	
Four double doses	42 (34.4%)	2 (20%)	
Female	89 (72.9%)	7 (70.0%)	1.000
Age (years)	41.2 ± 6.7	43.8 ± 7.1	0.240
Body mass index (kg/m^2^)	21.3 (19.7, 23.5)	22.7 (21.1, 25.7)	0.144
Creatinine clearance (mL/min)	84.0 ± 21.9	87.5 ± 27.8	0.640
CD4+ cell count	488 (373, 630)	443 (388, 635)	0.901
CD4+ cell count by category			0.207
CD4+ 201-350 cell/mm^3^	24 (19.7)	0 (0)	
CD4+ >350 cell/mm^3^	98 (80.4)	10 (100)	
Time elapsed since HIV diagnosis ≥3 months	80 (65.6)	10 (100)	0.030
Current cART			0.189
NNRTI based	109 (89.3%)	9 (90.0%)	
PI based	12 (9.8%)	0 (0%)	
Others	1 (0.8%)	1 (10.0%)	
Duration of cART (months)	82 (59-102)	86 (80-135)	0.284
History of drug resistance	8 (6.6%)	1 (10.0%)	0.519
HIV exposure category			1.000
Heterosexual	117 (95.9%)	10 (100%)	
Homosexual	2 (1.7%)	0 (0%)	
IVDU	1 (0.8%)	0 (0%)	
Blood transfusion	1 (0.8%)	0 (0%)	
Unknown	1 (0.8%)	0 (0%)	
Alcohol use			0.266
No	97 (79.5%)	6 (60.0%)	
Social drinking	20 (16.4%)	3 (30.0%)	
Regular drinking^a^	4 (3.3%)	1 (10.0%)	
Heavy drinking^b^	1 (0.8%)	0 (0.0%)	
Active smoking	9 (7.4%)	1 (10.0%)	0.558
Underlying diseases			0.967
Diabetes mellitus and IFG^c^	14 (11.5%)	2 (20.0%)	
Hypertension	19 (15.6%)	2 (20.0%)	
Dyslipidemia	18 (14.7%)	2 (20.0%)	
Others	19 (15.6%)	2 (20.0%)	

Data presented in number (%), means±SD, or median (IQR)

Abbreviation: cART, combination antiretroviral therapy; NNRTI, non-nucloside/nucleotide reverse transcriptase inhibitor; PI, protease inhibitor; IFG, impaired fasting glucose

^a^Defined as no more than 1 drink per day for women and no more than 2 drinks per day for men

^b^Defined as consuming an average of more than 1 drink per day for women and consuming an average of more than 2 drinks per day for men

^c^Defined as fasting plasma glucose from 5.6 mmol/L (100 mg/dL) to 6.9 mmol/L (125 mg/dL)

† Responder is defined as an antibody to hepatitis B surface antigen (anti-HBs) ≥10 mIU/ml

**Table 3:** Predictive factors from univariate analyses for high-level responders (anti HBs ≥100 mIU/ml) at month 7.

**Factors**	**High-Level Responders^†^ (n=105)**	**Low-level^‡^ & non-responder (n=27)**	**Unadjusted P-value**
Vaccination schedule			0.006
Standard dose	28 (26.7%)	16 (59.3%)	
Four single dose	37 (35.2%)	7 (25.9%)	
Four double doses	40 (38.1%)	4 (14.8%)	
Female	77 (73.3%)	19 (70.37%)	0.810
Age (years)	40.7 ± 6.1	44.0 ± 8.1	0.023
Body mass index (kg/m^2^)	21.3 (19.7-23.5)	21.7 (20.4-24.1)	0.453
Creatinine clearance (mL/min)	85.6 ± 22.4	79.1 ± 21.4	0.174
CD4+ cell count	493 (393-630)	476 (326-635)	0.441
CD4+ (%)	23 (19-28)	21 (17-25)	0.185
CD4+ cell count by category			0.267
CD4+ 201-350 cell/mm^3^	17 (16.2)	7 (25.9)	
CD4+ >350 cell/mm^3^	88 (83.8)	20 (74.1)	
Time elapsed since HIV diagnosis (months)	115 (83-170)	134 (83-188)	0.337
Current cART			0.092
NNRTI based	92 (87.6%)	26 (96.3%)	
PI based	12 (11.4%)	0 (0%)	
Others	1 (1.0%)	1 (3.7%)	
Duration of cART (months)	87 (59-103)	82 (65-84)	0.340
History of drug resistance	8 (7.6%)	1 (3.7%)	0.685
HIV exposure category			0.271
Heterosexual	102 (97.0%)	25 (92.6%)	
Homosexual	1 (1.0%)	1 (3.7%)	
IVDU	1 (1.0%)	0 (0%)	
Blood transfusion	0 (0%)	1 (3.7%)	
Unknown	1 (1.0%)	0 (0%)	
Alcohol use			0.857
No	83 (79.1%)	20 (74.1%)	
Social drinking	17 (16.2%)	6 (22.2%)	
Regular drinking^a^	4 (3.8%)	1 (3.7%)	
Heavy drinking^b^	1 (0.9%)	0 (0.0%)	
Active smoking	8 (7.6%)	2 (7.4%)	1.000
Underlying disease			0.297
Diabetes mellitus and IFG^c^	11 (10.5%)	5 (18.5%)	
Hypertension	16 (15.2%)	5 (18.5%)	
Dyslipidemia	14 (13.3%)	6 (20.2%)	
Others	16 (15.2%)	5 (18.5%)	

Data presented in number (%), means±SD, or median (IQR)

Abbreviation: cART, combination antiretroviral therapy; NNRTI, non-nucloside/nucleotide reverse transcriptase inhibitor; PI, protease inhibitor; IFG, impaired fasting glucose

^a^Defined as no more than 1 drink per day for women and no more than 2 drinks per day for men

^b^Defined as consuming an average of more than 1 drink per day for women and consuming an average of more than 2 drinks per day for men

^c^Defined as fasting plasma glucose from 5.6 mmol/L (100 mg/dL) to 6.9 mmol/L (125 mg/dL)

† High-Level Responders is defined as antibody to hepatitis B surface antigen (anti-HBs) ≥100 mIU/ml

‡ Low-level Responders is defined as antibody to hepatitis B surface antigen (anti-HBs) 10-99.9 mIU/ml

### Safety

The adverse events of each vaccination regimen are shown in Table 4. 57 of 132 participants (43.2%) reported at least one adverse event. The most common adverse events were pain at injection site (42.4%), fatigue (10.6%) and swelling at injection site (10.1%). The occurrence of pain at injection site was significantly more common in the Four double doses group comparing with the Standard doses group (*P*=0.017). There were no serious adverse events related to any vaccination regimen.

**Table 4:** Local and Systemic Adverse Events.

	**All (n=132)**	**Standard doses (n=44)**	**4 doses (n=44**)	**P-value^¶^**	**4 double doses (n=44**)	**P-value^†^**
Edema at injected site	14 (10.1%)	4 (9.1%)	7 (15.9%)	0.521	3 (6.8%)	1.000
Redness at injected site	7 (5.3%)	2 (4.5%)	2 (4.5%)	1.000	3 (6.8%)	1.000
Pain at injected site	56 (42.4%)	13 (29.5%)	18 (40.1%)	0.372	25 (56.8%)	0.017
Fever	8 (6.1%)	2 (4.5%)	3 (6.8%)	1.000	3 (6.8%)	1.000
Headache	8 (6.1%)	3 (6.8%)	1 (2.3%)	0.616	4 (9.1%)	1.000
Fatigue	14 (10.6%)	4 (9.1%)	2 (4.5%)	0.676	8 (18.2%)	0.352
Other symptoms	7 (5.3%)	2 (4.5%)	2 (4.5%)	1.000	3 (6.8%)	1.000

Data presented in number (%)

¶ Compare between the standard doses group and the 4 doses group

† Compare between the standard doses group and the 4 double doses group

## Discussion

Previous studies consistently showed that persons with HIV infection have an impaired response to HBV vaccine, with the response rates between 33.3% and 65% after standard HBV vaccination schedules [7–10]. In contrast, our study demonstrated that the percentage of responders to HBV vaccination in HIV-infected adults was 89% in those receiving the standard HBV vaccine regimen; this response rate is almost as high as that achieved in non-HIV healthy adults [11,12]. Many factors have been shown to have an impact on the response rate including a high CD4+ cell count [10,13–15], the use of cART [13], female sex [10,13,14], low or undetectable plasma HIV-1 RNA [10,13–15], younger age [10], non-smoking [10], and longer duration of cART [13]. The HIV-infected participants enrolled to our study had CD4+ cell counts of more than 200 cells/mm^3^, undetectable plasma HIV-1 RNA, and most were female. These factors, in particular the undetectable plasma HIV-1 RNA, may lead to the high response rate in our study. In the subgroup analysis of a study by Launay et al, the response rate of standard HBV vaccine regimen was 65% in participants with HIV infection who had CD4+ cell counts of more than 200 cells/mm^3^ and undetectable plasma HIV-1 RNA [10]. However, the response rate of standard HBV vaccine in this study was still lower than ours with similar subset of the HIV-positive population. Interestingly, a study from Thailand by Paitoonpong et al also found a considerably high response rate of 71.4% in Thai HIV-infected adults with undetectable plasma HIV-1 RNA [20]. This study had 60% of female, which was similar to our study, in contrast to 32% in the study by Launay et al [10]. This raises a possibility of gender, racial, and/or small body size influences on the vaccine response that Thai or Southeast Asian population may have a better response to standard HBV vaccination. Nonetheless, we failed to demonstrate that female gender and lower body mass index were associated with higher vaccine response rate. The only factor we found associated with higher response rate at month 7 from the univariate analysis was the shorter elapsed time from HIV diagnosis to antiretroviral therapy. It is uncertain for the reason as well as the clinical implication of this finding.

To improve the vaccine efficacy, several studies have found that increasing frequency and/or dosage of HBV vaccination can increase response rate in HIV-infected individuals [9,10,14,15,17]. A study by Fonseca et al showed that giving a double dosage of HBV vaccine statistically improved seroconversion only in HIV-infected adults with CD4+ cell count ≥350 cells/mm^3^ and low plasma HIV-1 RNA (64.3% vs. 39.3%; *P*=0.008) and they recommended to use a double dosage as a primary vaccination series when these criteria are met [15]. Our study confirmed that giving four double doses vaccination produced a higher response rates of 95.5% and 88.6% at month 7 and 12, compared to 88.6% and 70.4%, respectively, using the standard vaccination; we could only demonstrate a marginal statistically significant difference at month 12. However, doubling the vaccine doses may be problematic in clinical practice since it would also double the cost as well as adverse events. We, therefore, added another group that increased only the frequency of vaccination but used standard vaccine dosage (the Four doses group). Although the response rates in this Four doses group were higher than the standard doses group (93.2% vs. 88.6% at month 7 and 86.4% vs. 70.4% at month 12), these were not statistically significant (*P*=0.713 and 0.119, respectively).

Interestingly, the results of this study showed that increase frequency and dosage of HBV vaccine can increase the level of anti-HBs titers as well as the high-titer response rate (anti HBs ≥100 mIU/ml), in particular at month 12 of vaccination. This observation was similar to that in the study by Porsch et al [21]. However, the clinical significance of the higher antibody titer is still uncertain. Previous studies in immunocompetent individuals showed conflicting results regarding the high-titer response rate and the long-term immunogenicity of HBV vaccine. Some studies found that high anti-HBs titers after HBV vaccination are associated with lifelong immunity [14,22,23], but some showed that despite antibody decline or loss, immune memory exhibits long-term persistence, and booster doses of vaccine do not seem necessary to ensure long-term protection [24–26]. Until now, the importance of high anti-HBs titer after vaccination remains inconclusive, especially in immunocompromised hosts such as HIV-infected participants.

Our study also confirmed that HBV vaccine is safe and well tolerated in HIV-infected participants. Although the local adverse effects were more common with increased frequency and dosage of vaccine, the systemic and serious adverse events were extremely rare in our study as well as in other studies in HIV-infected population [10,15].

The strength of our study was the 100% retention rate with zero mortality rate. This may be explained by the intensive counselling by the study team, good patient-healthcare provider relationship, and participants’ realization on the importance of HBV vaccination as well as antiretroviral therapy. However, our study had some limitations. First, the study included only HIV-infected adults with CD4+ cell counts >200 cells/mm^3^ and undetectable plasma HIV-1 RNA. The results could not be applied to those with low CD4+ cell counts and detectable plasma HIV-1 RNA. A prospective study in these special situations may be needed. Second, we did not design the trial to compare the response rates between the Four doses group and the Four double doses group. Although we found similar response rates, the comparison of the results between these two groups may not be possible due to the inadequate sample size. A large multicenter, randomized controlled, non-inferiority trial is necessary to compare the response rates between these two vaccination strategies. Third, our study was designed to follow-up the participants until 1 year after the first dose of vaccination. As mentioned above, the importance of high anti HBs titers in HIV-infected participants remains uncertain; longer term of follow-up should be done to determine whether the participants with higher anti HBs titers have longer period of protection.

In conclusions, in northern Thailand, the standard HBV vaccination in HIV-infected adults with CD4+ cell counts >200 cells/mm^3^ and undetectable plasma HIV-1 RNA is highly effective. Regimens of four injections of either standard or double doses may yield small increase in the response rates and longer term of protection as well as can induce higher levels of antibody to the virus. However, the clinical significance of the higher antibody titer in HIV-infected individuals is uncertain.

## Supporting Information

Checklist S1 **CONSORT Checklist.**
(DOC)

Protocol S1 **Trial Protocol.**
(PDF)

Protocol S2 **English Translation of Trial Protocol.**
(DOCX)

## References

[1] Konopnicki D, Mocroft A, de Wit S, Antunes F, Ledergerber B et al. (2005) Hepatitis B and HIV: prevalence, AIDS progression, response to highly active antiretroviral therapy and increased mortality in the EuroSIDA cohort. AIDS 19: 593-601. doi:10.1097/01.aids.0000163936.99401.fe. PubMed: 15802978. 15802978

[2] Kellerman SE, Hanson DL, McNaghten AD, Fleming PL (2003) Prevalence of chronic hepatitis B and incidence of acute hepatitis B infection in human immunodeficiency virus-infected subjects. J Infect Dis 188: 571-577. doi:10.1086/377135. PubMed: 12898445. 12898445

[3] Salmon-Ceron D, Lewden C, Morlat P, Bévilacqua S, Jougla E et al. (2005) Liver disease as a major cause of death among HIV infected patients: role of hepatitis C and B viruses and alcohol. J Hepatol 42: 799-805. doi:10.1016/j.jhep.2005.01.022. PubMed: 15973779. 15973779

[4] Thio CL, Seaberg EC, Skolasky R Jr, Phair J, Visscher B et al. (2002) HIV-1, hepatitis B virus, and risk of liver-related mortality in the Multicenter Cohort Study (MACS). Lancet 360: 1921-1926. doi:10.1016/S0140-6736(02)11913-1. PubMed: 12493258. 12493258

[5] Kaplan JE, Benson C, Holmes KH, Brooks JT, Pau A et al. (2009) Guidelines for prevention and treatment of opportunistic infections in HIV-infected adults and adolescents: recommendations from CDC, the National Institutes of Health, and the HIV Medicine Association of the Infectious Diseases Society of America. MMWR Recomm Rep 58: 1-207; quiz CE201-204 19357635

[6] Geretti AM, Brook G, Cameron C, Chadwick D, Heyderman RS et al. (2008) British HIV Association guidelines for immunization of HIV-infected adults 2008. HIV Med 9: 795-848. doi:10.1111/j.1468-1293.2008.00637.x. PubMed: 18983477. 18983477

[7] Loke RH, Murray-Lyon IM, Coleman JC, Evans BA, Zuckerman AJ (1990) Diminished response to recombinant hepatitis B vaccine in homosexual men with HIV antibody: an indicator of poor prognosis. J Med Virol 31: 109-111. doi:10.1002/jmv.1890310207. PubMed: 2143776. 2143776

[8] da Mota Silveira Sasaki MG , Sobroza De Mello R , Focaccia Siciliano R , Wang Lda Mota Silveira Sasaki MG, Sobroza De Mello R, Focaccia Siciliano R, Wang L (1998) Response of HIV/AIDS Patients to Hepatitis B Recombinant Vaccine. Braz J Infect Dis 2: 236-240. PubMed: 11103014. 11103014

[9] Kalinowska-Nowak A, Bociaga-Jasik M, Garlicki A, Mach T (2007) [Efficacy of vaccination against hepatitis B in adult with HIV infection]. Przegl Epidemiol 61: 339-347. PubMed: 17956052. 17956052

[10] Launay O, van der Vliet D, Rosenberg AR, Michel ML, Piroth L et al. (2011) Safety and immunogenicity of 4 intramuscular double doses and 4 intradermal low doses vs standard hepatitis B vaccine regimen in adults with HIV-1: a randomized controlled trial. JAMA 305: 1432-1440. doi:10.1001/jama.2011.351. PubMed: 21486976. 21486976

[11] André FE (1989) Summary of safety and efficacy data on a yeast-derived hepatitis B vaccine. Am J Med 87: 14S-20S. doi:10.1016/0002-9343(89)90525-1. PubMed: 2528292. 2528292

[12] Zajac BA, West DJ, McAleer WJ, Scolnick EM (1986) Overview of clinical studies with hepatitis B vaccine made by recombinant DNA. J Infect 13 Suppl A: 39-45. doi:10.1016/S0163-4453(86)92668-X. PubMed: 2943814. 2943814

[13] de Vries-Sluijs TE, Hansen BE, van Doornum GJ, Kauffmann RH, Leyten EM et al. (2011) A randomized controlled study of accelerated versus standard hepatitis B vaccination in HIV-positive patients. J Infect Dis 203: 984-991. doi:10.1093/infdis/jiq137. PubMed: 21266513. 21266513

[14] Cruciani M, Mengoli C, Serpelloni G, Lanza A, Gomma M et al. (2009) Serologic response to hepatitis B vaccine with high dose and increasing number of injections in HIV infected adult patients. Vaccine 27: 17-22. doi:10.1016/j.vaccine.2008.10.040. PubMed: 18984022. 18984022

[15] Fonseca MO, Pang LW, de Paula Cavalheiro N, Barone AA, Heloisa Lopes M (2005) Randomized trial of recombinant hepatitis B vaccine in HIV-infected adult patients comparing a standard dose to a double dose. Vaccine 23: 2902-2908. doi:10.1016/j.vaccine.2004.11.057. PubMed: 15780739. 15780739

[16] Soriano V, Puoti M, Bonacini M, Brook G, Cargnel A et al. (2005) Care of patients with chronic hepatitis B and HIV co-infection: recommendations from an HIV-HBV International Panel. AIDS 19: 221-240. doi:10.1097/01.aids.0000192093.46506.e5. PubMed: 15718833. 15718833

[17] Rey D, Krantz V, Partisani M, Schmitt MP, Meyer P et al. (2000) Increasing the number of hepatitis B vaccine injections augments anti-HBs response rate in HIV-infected patients. Effects on HIV-1 viral load. Vaccine 18: 1161-1165. doi:10.1016/S0264-410X(99)00389-8. PubMed: 10649616. 10649616

[18] the Royal College of Physicians of; Thailand. Recommended Adult and Elderly Immunization Schedule (In Thai) (2012). Available: http://www.rcpt.org/index.php/2012-10-03-16-53-39/category/6-2013-02-02-09-02-52.html?download=56%3A2013-02-02-09-08-53. Accessed 30 January 2013.

[19] Pugh RN, Murray-Lyon IM, Dawson JL, Pietroni MC, Williams R (1973) Transection of the oesophagus for bleeding oesophageal varices. Br J Surg 60: 646-649. doi:10.1002/bjs.1800600817. PubMed: 4541913. 4541913

[20] Paitoonpong L, Suankratay C (2008) Immunological response to hepatitis B vaccination in patients with AIDS and virological response to highly active antiretroviral therapy. Scand J Infect Dis 40: 54-58. doi:10.1080/00365540701522975. PubMed: 17852939. 17852939

[21] Potsch DV, Camacho LA, Tuboi S, Villar LM, Miguel JC et al. (2012) Vaccination against hepatitis B with 4-double doses increases response rates and antibodies titers in HIV-infected adults. Vaccine 30: 5973-5977. doi:10.1016/j.vaccine.2012.07.028. PubMed: 22828589. 22828589

[22] Hadler SC, Francis DP, Maynard JE, Thompson SE, Judson FN et al. (1986) Long-term immunogenicity and efficacy of hepatitis B vaccine in homosexual men. N Engl J Med 315: 209-214. doi:10.1056/NEJM198607243150401. PubMed: 2941687. 2941687

[23] Gesemann M, Scheiermann N (1995) Quantification of hepatitis B vaccine-induced antibodies as a predictor of anti-HBs persistence. Vaccine 13: 443-447. doi:10.1016/0264-410X(94)00010-K. PubMed: 7639012. 7639012

[24] Zanetti AR, Mariano A, Romanò L, D'Amelio R, Chironna M et al. (2005) Long-term immunogenicity of hepatitis B vaccination and policy for booster: an Italian multicentre study. Lancet 366: 1379-1384. doi:10.1016/S0140-6736(05)67568-X. PubMed: 16226616. 16226616

[25] Van Damme P, Van Herck K (2007) A review of the long-term protection after hepatitis A and B vaccination. Travel Med Infect Dis 5: 79-84. doi:10.1016/j.tmaid.2006.04.004. PubMed: 17298912. 17298912

[26] Gabbuti A, Romanò L, Blanc P, Meacci F, Amendola A et al. (2007) Long-term immunogenicity of hepatitis B vaccination in a cohort of Italian healthy adolescents. Vaccine 25: 3129-3132. doi:10.1016/j.vaccine.2007.01.045. PubMed: 17291637. 17291637

