# Supplementary material for: Comparison of Immunogenicity and Safety of Four Doses and Four Double Doses vs. Standard Doses of Hepatitis B Vaccination in HIV-Infected Adults: A Randomized, Controlled Trial
Source: PLoS One. 2013 Nov 12;8(11):e80409. doi: 10.1371/journal.pone.0080409 (PMC3827227; doi:10.1371/journal.pone.0080409)
Supplement: Protocol S1 — Trial Protocol. (PDF) [file pone.0080409.s002.pdf]

ข้อเสนอโครงการวิจัย (Research Proposal)

ฉบับรับรอง
การวิจัย
วันที่ 14 DEC 10

1. ชื่อโครงการ (Title of the project)

(ภาษาไทย) การศึกษาแบบสุ่มเพื่อศึกษาประสิทธิภาพในการกระตุ้นให้เกิดภูมิคุ้มกันต่อวัคซีนไวรัสตับอักเสบบีโดยการเพิ่มขนาดและความถี่ในการฉีดวัคซีนในผู้ป่วยที่ติดเชื้อเอชไอวี

(ภาษาอังกฤษ) Efficacy of modified recombinant hepatitis B vaccination schedule in HIV- infected adults subjects : A randomized control trial

2. สาขาที่ทำวิจัย

อายุรศาสตร์โรคติดเชื้อ

3.รายนามผู้ทำการวิจัย

แพทย์หญิงกนกพร ไชยกลาง	แพทย์ผู้วิจัย
ดร.จิรประภา วิภาษา	นักวิจัย
ศาสตราจารย์นายแพทย์ขวัญชัย ศุภรัตน์ภิญโญ	อาจารย์ที่ปรึกษา
ผู้ช่วยศาสตราจารย์แพทย์หญิงรมณีย์ ชัยวาฤทธิ์	ผู้ร่วมวิจัย
อาจารย์นายแพทย์นนทกานต์ นันทจิต	ผู้ร่วมวิจัย

4. วัตถุประสงค์การวิจัย (Research objectives)

4.1วัตถุประสงค์หลักเป็นการศึกษาแบบสุ่มเพื่อเปรียบเทียบประสิทธิภาพในการกระตุ้นให้เกิดภูมิคุ้มกันต่อวัคซีนไวรัสตับอักเสบบีโดยการเพิ่มความถี่ในการฉีดวัคซีนและการเพิ่มขนาดและความถี่ในการฉีดวัคซีนเปรียบเทียบกับการฉีดวัคซีนไวรัสตับอักเสบบีตามขนาดและความถี่มาตรฐานในผู้ป่วยที่ติดเชื้อเอชไอวี

4.2 วัตถุประสงค์รอง

4.2.1 เพื่อศึกษาลักษณะ (phenotype) ของ B cells ในการตอบสนองต่อวัคซีนตับอักเสบบีในผู้ติดเชื้อเอชไอวีที่ได้รับวัคซีนขนาดต่าง ๆ และเปรียบเทียบกับคนปกติซึ่งเป็นกลุ่มควบคุม

4.2.2 เพื่อศึกษาระดับของ interleukin (IL)-6, IL-10 และ IL-21 ในพลาสมาหลังได้รับวัคซีน

4.2.3 เพื่อศึกษาระดับของแอนติบอดี คุณสมบัติของ memory B cells ในการสร้างแอนติบอดีที่จำเพาะต่อไวรัสตับอักเสบบีที่เวลาต่าง ๆ หลังได้รับวัคซีน

4.2.4 เพื่อศึกษาการตอบสนองของภูมิคุ้มกันระบบเซลล์ (cell-mediated immunity) ภายหลังการฉีดวัคซีน

4.2.5 เพื่อศึกษาระดับการคงอยู่ของภูมิคุ้มกันต่อวัคซีนไวรัสตับอักเสบบีหลังจากฉีดวัคซีนเข็มสุดท้าย 6 เดือน

5. ความเป็นมาหรือทบทวนเรื่องราวเดิม (Background or Literature review)

ในผู้ป่วยที่ติดเชื้อเอชไอวีพบว่ามีการตอบสนองทางภูมิคุ้มกันหลังจากฉีดวัคซีนไวรัสตับอักเสบบีน้อยกว่าในผู้ที่สุขภาพร่างกายแข็งแรงดีที่ไม่ติดเชื้อเอชไอวี การศึกษาของ Da Moto และคณะพบว่าในผู้ป่วยติดเชื้อเอชไอวีพบว่ามีเพียง 45.5% เท่านั้นที่มีภูมิคุ้มกันต่อไวรัสตับอักเสบบีหลังจากฉีดวัคซีนตามตารางมาตรฐาน (20 μg ที่ 0, 1, 6 เดือน) เปรียบเทียบกับอาสาสมัครที่ไม่ติดเชื้อเอชไอวีพบว่ามีภูมิคุ้มกันทั้งหมด 100% หลังฉีดวัคซีน(da Mota Silveira Sasaki et al., 1998) และการศึกษาของ Kalinowska-Nowak และคณะพบว่าหลังจากฉีดวัคซีนไวรัสตับอักเสบบีในผู้ป่วยเอชไอวี พบว่ามีภูมิคุ้มกันเพียง 63% หลังจากฉีดวัคซีนครบสามเข็ม(Kalinowska-Nowak et al., 2007)

ได้มีความพยายามในการศึกษาเกี่ยวกับการปรับปรุงวิธีการฉีดวัคซีนไวรัสตับอักเสบบีในผู้ป่วยเอชไอวีเพื่อเพิ่มการกระตุ้นให้เกิดภูมิคุ้มกันเช่น การฉีดวัคซีนให้ถี่มากขึ้น (Rey et al., 2000) การเพิ่มขนาดของวัคซีนให้สูงขึ้น(Fonseca et al., 2005) รวมถึงการใช้ไซโตคายด์หรือสารกระตุ้นภูมิคุ้มกันอื่นร่วมกับการฉีดวัคซีนไวรัสตับอักเสบบี (Cooper et al., 2008; Sasaki et al., 2003) การศึกษาที่ทำเกี่ยวกับการเพิ่มการฉีดวัคซีนให้ถี่มากขึ้นโดย Rey และคณะพบว่าหลังจากฉีดวัคซีนไวรัสตับอักเสบบีขนาด 20 μg ที่ 0,1,2 เดือน พบว่ามีภูมิคุ้มกันเพียง 55% หลังจากฉีดวัคซีนครบสามเข็มแรกและในกลุ่มที่ไม่ตอบสนองต่อการฉีดวัคซีนรอบแรกก็นำมาฉีดวัคซีนซ้ำอีก 3 เข็ม พบว่ามีการตอบสนองทางภูมิคุ้มกันเพิ่มเป็น 90% (Rey et al., 2000) การศึกษาของ Kalinowska-Nowak และคณะพบว่าหลังจากฉีดวัคซีนไวรัสตับอักเสบบีในผู้ป่วยเอชไอวีพบว่ามีภูมิคุ้มกันเพียง 63% หลังจากฉีดวัคซีนครบสามเข็ม และในผู้ป่วยที่ไม่มีภูมิคุ้มกันหลังจากฉีดวัคซีนครบสามเข็ม เมื่อนำมาฉีดวัคซีนกระตุ้นเพิ่มอีกหนึ่งเข็มพบว่าผู้ป่วยมีภูมิคุ้มกันเพิ่มเป็น 79.7% และ 87.1% หลังจากฉีดวัคซีนกระตุ้นเข็มที่สองและ 90.7% หลังจากฉีดวัคซีนกระตุ้นเข็มที่สาม (Kalinowska-Nowak et al., 2007) ซึ่งการศึกษาทั้งสองนี้แสดงให้เห็นว่าการเพิ่มความถี่ของการฉีดวัคซีนสามารถกระตุ้นให้เกิดภูมิคุ้มกันที่มากขึ้นได้

การศึกษาที่ทำเกี่ยวกับการเพิ่มขนาดวัคซีน เป็นการศึกษาในประเทศบราซิลทำโดย Oliveira และคณะโดยได้ทำการศึกษาแบบสุ่มเปรียบเทียบการฉีดวัคซีนขนาด 40 μg และ 20 μg โดยฉีดที่ 0,1,6 เดือน พบว่ามีอาสาสมัครที่มีภูมิคุ้มกันต่อไวรัสตับอักเสบ (Anti-HBs $\geq$ 10 mIU/mL) เท่ากับ 47% และ 34% ตามลำดับ (p=0.07) แต่ถ้าดูเฉพาะในกลุ่มที่ CD4 $\geq$ 350 cell/mm^3 พบว่าในกลุ่มที่ได้วัคซีนขนาดสองเท่ามีภูมิคุ้มกัน 64.3% เทียบกับในกลุ่มที่ได้วัคซีนขนาดปกติมีภูมิคุ้มกัน 39.3% (p=0.008) และในกลุ่มที่ viral

load < 10,000 copies/ml พบว่าในกลุ่มที่ได้วัคซีนขนาดสองเท่ามีภูมิคุ้มกัน 58.3% เทียบกับในกลุ่มที่ได้วัคซีนขนาดปกติมีภูมิคุ้มกัน 37.3% (p=0.01) การศึกษานี้แสดงให้เห็นว่าการเพิ่มขนาดของวัคซีนไวรัสตับอักเสบบีเป็นสองเท่าจะสามารถเพิ่มระดับของภูมิคุ้มกันได้ในผู้ป่วยเอชไอวีที่มี CD4 ≥ 350 cell/mm^3 และ viral load < 10,000 copies/ml โดยพบว่าการเกิดอาการไม่พึงประสงค์จากการฉีดวัคซีนไม่แตกต่างกันในทั้งสองกลุ่ม และไม่พบอาการไม่พึงประสงค์รุนแรงจากการให้วัคซีน การศึกษาโดย Pasricha และคณะทำการศึกษาในประเทศอินเดียได้ทำการศึกษาการฉีดวัคซีนไวรัสตับอักเสบบีขนาด 40 µg ที่ 0,1,6 เดือน โดยแบ่งผู้ป่วยเป็นสองกลุ่มคือกลุ่มที่ CD4 < 200 cell/mm^3 และกลุ่ม CD4 ≥ 200 cell/mm^3 พบว่าในกลุ่ม CD4 ≥ 200 cell/mm^3 หลังจากฉีดวัคซีนครบสามเข็มมีการตอบสนองทางภูมิคุ้มกัน 100% ส่วนในกลุ่ม CD4 < 200 cell/mm^3 พบว่ามีการตอบสนองทางภูมิคุ้มกันเพียง 47% ซึ่งการศึกษานี้แสดงให้เห็นว่าระดับของ CD4 มีผลต่อการเพิ่มขึ้นของระดับภูมิคุ้มกัน (Pasricha et al., 2006) แต่ก็มีการศึกษาที่ผลการศึกษาออกมาพบว่าการเพิ่มขนาดของวัคซีนไวรัสตับอักเสบบีไม่เพิ่มการตอบสนองของภูมิคุ้มกันโดยเป็นการศึกษาของ Cornejo-Juarez และคณะทำการศึกษาในประเทศเม็กซิโก ได้ทำการศึกษาเปรียบเทียบการฉีดวัคซีนไวรัสตับอักเสบบีขนาด 10 µg และ 40 µg ที่ 0,1,6 เดือน พบว่าทั้งสองกลุ่มมีภูมิคุ้มกันขึ้นสูงหลังจากฉีดวัคซีนแต่ไม่มีความแตกต่างกันในการเพิ่มขึ้นของภูมิคุ้มกันในผู้ป่วยทั้งสองกลุ่ม คือในกลุ่มที่ได้รับวัคซีน 10 µg มีภูมิคุ้มกัน 61.5% เทียบกับกลุ่มที่ได้รับวัคซีน 40 µg มีภูมิคุ้มกัน 60% (p=0.889) และพบว่าในกลุ่มที่ CD4 ≥ 200 cell/mm^3 มีการเพิ่มของภูมิคุ้มกันมากกว่าในกลุ่มที่ CD4 < 200 cell/mm^3 อย่างมีนัยสำคัญทางสถิติคือเท่ากับ 86.8% และ 36.6% ตามลำดับ (p=0.003) พบว่าการฉีดวัคซีนไวรัสตับอักเสบบีผู้ป่วยสามารถทนได้ดี มีผู้ป่วยหนึ่งคนรายงานว่ามีอาการปวดบริเวณที่ฉีดวัคซีนและอีกหนึ่งคนรายงานว่ามีอาการปวดและแดงบริเวณที่ฉีดวัคซีน ไม่พบว่ามีการรายงานถึงผลข้างเคียงที่รุนแรงที่เกี่ยวกับการฉีดวัคซีน การศึกษานี้สรุปว่าการเพิ่มขนาดของวัคซีนไวรัสตับอักเสบบีไม่เพิ่มการตอบสนองของภูมิคุ้มกัน แต่ปัจจัยที่มีผลต่อการเพิ่มการตอบสนองต่อวัคซีนคือระดับของ CD4 ที่มากกว่า 200 cell/mm^3 (Cornejo-Juarez et al., 2006)

การศึกษาเกี่ยวกับการใช้ไซโตคายด์หรือสารกระตุ้นภูมิคุ้มกันอื่นร่วมกับการเพิ่มขนาดวัคซีนไวรัสตับอักเสบบี โดยเป็นการศึกษาของ Das Gracas Sasaki และคณะได้ทำการศึกษาในประเทศบราซิล เป็นการศึกษาแบบสุ่มโดยการให้ GM-CSF 20 µg ร่วมกับการฉีดวัคซีนไวรัสตับอักเสบบีขนาด 40 µg ที่ 0,1,6 เดือน ผลการศึกษาพบว่าหลังจากฉีดวัคซีนเข็มแรก การตอบสนองทางภูมิคุ้มกันไม่มีความแตกต่างกันคือ 30% ในกลุ่มที่ได้รับ GM-CSF 20 µg และ 15 % ในกลุ่มควบคุม แต่หลังจากฉีดวัคซีนไวรัสตับอักเสบเข็มที่สองพบว่าในกลุ่มที่ได้รับ GM-CSF 20 µg ร่วมกับการฉีดวัคซีนไวรัสตับอักเสบบีขนาด 40 µg มีการตอบสนองทางภูมิคุ้มกันสูงกว่ากลุ่มควบคุมอย่างมีนัยสำคัญทางสถิติคือ 62% และ 30% ตามลำดับ (P<0.0074) แต่หลังจากฉีดวัคซีนครบสามเข็มก็พบว่าไม่มีความแตกต่างกันในการตอบสนองทางภูมิคุ้มกันคือ 72% ในกลุ่มที่ได้รับ GM-CSF 20 µg และ 60 % ในกลุ่มควบคุม ซึ่งการให้วัคซีนไวรัสตับอักเสบบีขนาด 40 µg ผู้ป่วยสามารถทนได้ดี มีผู้ป่วย 25% ที่รายงานว่ามีอาการปวดเล็กน้อยบริเวณที่ฉีดวัคซีนและไม่พบว่ามีการรายงานถึงผลข้างเคียงที่รุนแรงที่เกี่ยวกับการฉีดวัคซีนหรือการให้ GM-CSF (Das Gracas Sasaki et al.,

2003) การศึกษาโดย Cooper และคณะโดยเป็นการศึกษาแบบสุ่ม ได้ทำการศึกษาโดยการฉีด CpG oligodeoxynucleotide (CPG 7909) ซึ่งเป็นสารกระตุ้นภูมิคุ้มกันร่วมกับการฉีดวัคซีนไวรัสตับอักเสบบี ขนาด 40 μg ที่ 0,1,2 เดือน ผลการศึกษาพบว่าในกลุ่มที่ได้รับ CPG 7909 ร่วมกับการฉีดวัคซีนไวรัสตับอักเสบบีขนาด 40 μg พบว่ามีการตอบสนองทางภูมิคุ้มกันและการคงอยู่ของระดับภูมิคุ้มกันมากกว่าในกลุ่มที่ไม่ได้รับ CPG 7909 อย่างมีนัยสำคัญทางสถิติ ($P<0.05$) (Cooper et al., 2008)

ต่อมาได้มีการศึกษาทั้งการเพิ่มขนาดและความถี่ของไวรัสตับอักเสบบีในผู้ป่วยเอชไอวี แต่เป็นการศึกษาแบบไปข้างหน้า โดยเป็นการศึกษาของ Mario และคณะโดยทำการศึกษาในประเทศอิตาลีโดยได้ทำการฉีดวัคซีนไวรัสตับอักเสบบีขนาดสองเท่า (40 μg) ที่ 0,1,2 เดือนและในผู้ป่วยที่ไม่มีการตอบสนองทางภูมิคุ้มกันหลังจากฉีดวัคซีนรอบแรกก็จะมีการฉีดวัคซีนซ้ำอีก 1-3 เข็ม หลังจากฉีดวัคซีนครบสามเข็มแรกพบว่าอาสาสมัครมีภูมิคุ้มกันต่อไวรัสตับอักเสบบี 60% และ 89.2% หลังจากให้วัคซีนซ้ำ และไม่พบว่ามีผลข้างเคียงใดๆ ที่เกี่ยวข้องกับการฉีดวัคซีน ซึ่งการศึกษานี้แสดงให้เห็นว่าการเพิ่มขนาดและความถี่ของการฉีดวัคซีนสามารถกระตุ้นให้เกิดภูมิคุ้มกันเพิ่มขึ้นได้(Cruciani et al., 2009)

เนื่องจากการศึกษาที่ผ่านมาแสดงให้เห็นว่าการเพิ่มความถี่ของวัคซีนไวรัสตับอักเสบบีสามารถช่วยกระตุ้นให้เกิดภูมิคุ้มกันที่ดีขึ้นได้ แต่การเพิ่มขนาดของวัคซีนไวรัสตับอักเสบบีมีทั้งข้อมูลที่สนับสนุนและขัดแย้ง นอกจากนี้ยังมีปัจจัยอื่นๆ ที่มีผลต่อการตอบสนองทางภูมิคุ้มกันเช่น ระดับ CD4 ปัจจัยทางพันธุกรรมและอาจจะมีปัจจัยทางเชื้อชาติเข้ามาเกี่ยวข้องด้วย

6. หลักการและเหตุผล (Rationale)

การติดเชื้อเอชไอวีและการติดเชื้อไวรัสตับอักเสบบีมีปัจจัยเสี่ยงและทางติดต่อร่วมกัน ส่งผลให้มีอุบัติการณ์ของการติดเชื้อร่วมกันสูง (Denis et al., 1997; Francisci et al., 1995; Mandelli et al., 1988; Mendes-Correa et al., 2000; Ockenga et al., 1997; Scharschmidt et al., 1992; Treitinger et al., 1999) การติดเชื้อเอชไอวีร่วมกับการติดเชื้อไวรัสตับอักเสบบีจะทำให้เพิ่มโอกาสของการเป็นไวรัสตับอักเสบบีเรื้อรัง (Bodsworth et al., 1991; Hadler et al., 1991; Sinicco et al., 1997) มีการเพิ่มปริมาณของไวรัสตับอักเสบบีมากขึ้น (Colin et al., 1999; Gilson et al., 1997; Horvath and Raffanti, 1994; Krogsgaard et al., 1987) และเพิ่มโอกาสการแพร่เชื้อไปสู่บุคคลอื่นมากขึ้น ในปัจจุบันได้มียาต้านไวรัสเอชไอวีที่มีประสิทธิภาพสูง ทำให้ผู้ป่วยเอชไอวีมีอายุขัยยืนยาวขึ้น เสียชีวิตจากโรคติดเชื้อฉวยโอกาสลดลงแต่กลับพบว่าผู้ป่วยเอชไอวีเสียชีวิตจากโรคที่ไม่ได้เกิดจากโรคติดเชื้อมากขึ้น ซึ่งหนึ่งในสาเหตุการตายนั้นคือการเสียชีวิตจากโรคตับซึ่งมักจะสัมพันธ์กับการติดเชื้อไวรัสตับอักเสบบีและตับอักเสบซีเรื้อรัง (Lewden et al., 2005; Salmon-Ceron et al., 2005)

ตามคำแนะนำของ CDC ปี 2009 ให้คำแนะนำว่าควรจะต้องทำการฉีดวัคซีนไวรัสตับอักเสบบีให้กับผู้ป่วยติดเชื้อเอชไอวีทุกรายที่ยังไม่มีภูมิคุ้มกัน (คำแนะนำ AII) (Kaplan et al., 2009) ในคนปกติที่สุขภาพร่างกายแข็งแรงดี เมื่อทำการฉีดวัคซีนไวรัสตับอักเสบบีครบ 3 เข็มจะมีภูมิคุ้มกันต่อไวรัสตับอักเสบ

บีมากกว่า 90% (Andre, 1989; Zajac et al., 1986) ซึ่งแตกต่างจากผู้ป่วยที่ติดเชื้อเอชไอวีซึ่งมีความบกพร่องทางภูมิคุ้มกันจะมีการตอบสนองต่อการฉีดวัคซีนไวรัสตับอักเสบบีอยู่ที่ประมาณ 33-63 % (da Mota Silveira Sasaki et al., 1998; Kalinowska-Nowak et al., 2007; Loke et al., 1990) โดยปัจจัยอื่นๆ ที่มีผลต่อการตอบสนองที่ลดลงต่อวัคซีนไวรัสตับอักเสบบีได้แก่ อายุ การสูบบุหรี่ ความอ้วน และปัจจัยทางพันธุกรรม (Alper et al., 1989; Averhoff et al., 1998; Shaw et al., 1989; Weber et al., 1985; Wood et al., 1993) ซึ่งในปัจจุบันนี้ยังไม่มีข้อมูลเพียงพอเกี่ยวกับตารางการฉีดวัคซีนตับอักเสบบีที่เหมาะสมในผู้ป่วยที่ติดเชื้อเอชไอวี CDC ปี 2009 ให้คำแนะนำว่าในผู้ป่วยติดเชื้อเอชไอวีที่ยังไม่มีภูมิคุ้มกันต่อไวรัสตับอักเสบบีให้ฉีดวัคซีนขนาด 20 μg ที่ 0,1,6 เดือน (คำแนะนำ AII) และให้เจาะดูระดับของภูมิคุ้มกันต่อไวรัสตับอักเสบบีหลังจากฉีดยาเข็มสุดท้าย 1 เดือน (คำแนะนำ BIII) ถ้าพบว่ายังไม่มีภูมิคุ้มกันต่อไวรัสตับอักเสบบีให้ฉีดกระตุ้นรอบที่สองใหม่ (คำแนะนำ BIII) (Kaplan et al., 2009) คำแนะนำของ HIV-HBV International panel แนะนำว่าในผู้ป่วยติดเชื้อเอชไอวีที่ CD4 มากกว่า 500 cell/mm^3 ให้ฉีดวัคซีนตามตารางปกติ (20 μg ที่ 0,1,6 เดือน) ส่วนในผู้ป่วยที่ CD4 อยู่ระหว่าง 200-500 cell/mm^3 ให้ฉีดวัคซีนขนาด 20 μg ที่ 0,1,12 เดือน และในผู้ป่วยที่ฉีดวัคซีนแล้วไม่พบว่ามีภูมิคุ้มกันให้ฉีดวัคซีนซ้ำหรือฉีดใหม่ด้วยขนาด 40 μg ที่ 0,1,2 และ 6-12 เดือนจนกว่าจะพบว่ามีภูมิคุ้มกัน (Soriano et al., 2005) คำแนะนำของประเทศบราซิลให้ฉีดวัคซีนไวรัสตับอักเสบบีในขนาดและความถี่ที่มากขึ้นคือ 40 μg ที่ 0,1,2,6 เดือน (Ministério da Saúde do Brasil, 2002) ซึ่งเป็นขนาดและความถี่ที่แนะนำให้ฉีดแก่ผู้ป่วยไตวายระยะสุดท้ายที่ฟอกไตเป็นประจำ (2001; Bruguera et al., 1989)

เนื่องจากเชื้อ HIV อาศัยอยู่ใน CD4 T cells ซึ่งมีความสำคัญอย่างมากในระบบภูมิคุ้มกันรวมถึงบทบาทในการช่วยให้ B cells สร้างแอนติบอดี ผู้ติดเชื้อเอชไอวีจึงอาจมีการตอบสนองของภูมิคุ้มกันระบบสารน้ำ (Humoral immunity) ผิดปกติ ดังจะเห็นได้จากรายงานว่า humoral immunity ของผู้ติดเชื้อเอชไอวีต่อเชื้อไวรัสหลายชนิดเช่น measle, mumps, rubella, vericella zoster virus และไวรัสตับอักเสบบี (Bekker et al., 2006; Cruciani et al., 2009) B cells เป็นสิ่งจำเป็นต่อภูมิคุ้มกันระบบสารน้ำเนื่องจากเป็นเซลล์ต้นกำเนิดของแอนติบอดี โดยทั่วไป หลังจาก B cells จับกับแอนติเจนที่จำเพาะผ่าน receptor บนผิวเซลล์แล้ว B cells จะเคลื่อนที่ไปยังต่อมน้ำเหลืองบริเวณรอยต่อระหว่าง B cell follicle และ T cell area โดยขึ้นอยู่กับ chemokine ที่สร้างจากเซลล์อื่น ๆ (Okada et al., 2005) ในภาวะที่ได้รับสัญญานช่วยเหลืออย่างเพียงพอจาก CD4 T cells B cells จะเปลี่ยนแปลงคุณสมบัติไปเป็น short-lived plasma cells หรือเคลื่อนที่ไปยัง B cell follicle แบ่งตัวเกิดเป็น germinal centre (Tarlinton, 2006) สำหรับ B cells ที่ผ่านกระบวนการ somatic hypermutation จะเปลี่ยนไปเป็น plasma cells หรือ memory B cells แต่หากไม่สามารถจับกับแอนติเจนได้ดีหรือไม่ได้รับการช่วยเหลือจาก CD4 T cells อย่างเพียงพอ B cells เหล่านี้จะตายลงด้วยขบวนการ apoptosis จากนั้น plasma cells บางส่วนจะเคลื่อนที่ไปยังไขกระดูกทำหน้าที่สร้างแอนติบอดีได้ยาวนานเป็นเวลาหลายปี (Manz et al., 2005) ในทางทฤษฎีแล้ว การติดเชื้อหรือการฉีดวัคซีนควรชักนำให้เกิดทั้ง long-lived plasma cells เพื่อทำหน้าที่สร้างแอนติบอดีอย่างต่อเนื่องและ memory B cells ที่สามารถเปลี่ยนคุณสมบัติไป

เป็น plasma cells ได้อีกเมื่อมีการติดเชื้อซ้ำ ในระยะหลังนี้ มีรายงานเพิ่มมากขึ้นว่า long-lived plasma cells ซึ่งมีชีวิตอยู่นานและทำหน้าที่ผลิตแอนติบอดีในไขกระดูกและ memory B cells ที่ไหลเวียนอยู่ในระบบเลือดนั้นถูกควบคุมด้วยกลไกที่เป็นอิสระต่อกัน นอกจากนั้นระดับของแอนติบอดีหรือ memory B cells เพียงอย่างเดียวอาจไม่ได้บ่งบอกถึงการตอบสนองของภูมิคุ้มกันระบบสารน้ำทั้งหมด (Alwayn et al., 2001; Amanna et al., 2007; Anolik et al., 2004; DiLillo et al., 2008; Vallerskog et al., 2007)

ในสภาวะที่มีการอักเสบนานๆ interferon (IFN)-γ จะชักนำให้ plasma cells มีการแสดงออกของ chemokine receptor - CXCR3 - บนผิวเซลล์เพื่อเคลื่อนที่ไปยังบริเวณที่มีการอักเสบและเกิดการสร้างแอนติบอดีเพื่อกำจัดเชื้อ(Muehlinghaus et al., 2005) เมื่อการกำจัดเชื้อหรือการอักเสบสิ้นสุดลง ปัจจัยที่จำเป็นต่อการอยู่รอดของ short-lived plasma cells จะหมดลงไปด้วยทำให้เซลล์เหล่านี้ตาย ดังนั้นการถูกกระตุ้นด้วยแอนติเจนซ้ำ ๆ เช่นการติดเชื้อซ้ำหรือการติดเชื้อเรื้อรังมีการอักเสบซ้ำ ๆ อาจทำให้เกิด short-lived plasma cells ในทางตรงข้ามหากไม่มีการกระตุ้นอย่างต่อเนื่องจากแอนติเจนหรือไม่มีการอักเสบเรื้อรัง plasma cells จะมีการแสดงออกของ CXCR4 ทำให้เซลล์เหล่านี้เคลื่อนที่ไปยังไขกระดูกมีชีวิตอยู่ได้เป็นเวลานาน (Tokoyoda et al., 2004) การเปลี่ยนแปลงของ activated B cells ไปเป็น long-lived plasma cells นั้นขึ้นอยู่กับหลายปัจจัยเช่น cytokines (Interleukin[IL]-6 และ IL-21) และการแสดงออกของ transcription factor เช่น B-lymphocyte-induced maturation protein 1(BLIMP1) (Moser et al., 2006) และ X-box binding protein-1 (XBP-1) (Iwakoshi et al., 2003) มีรายงานว่า B cells จากผู้ติดเชื้อเอชไอวีมี FCRL4+ จะมีการแสดงออกของ CXCR3 และเซลล์เหล่านี้มีการตอบสนองต่ำ (Moir et al., 2008) จึงอาจเป็นไปได้ว่าการตอบสนองของแอนติบอดีและ memory B cells ในกลุ่มผู้ติดเชื้อเอชไอวีอาจมีอายุไม่นาน ลักษณะของ B cells กลุ่มต่าง ๆ สามารถแบ่งได้โดยอาศัยเครื่องหมายบนผิวเซลล์ดังแสดงในตารางที่ 1

ตารางที่ 1 ลักษณะของ B cells กลุ่มต่าง ๆ แบ่งตามเครื่องหมายบนผิวเซลล์

	CD19	CD10[a]	CD21	CD27[b]	CD38	CD138	IgG
Naïve B cells	+	-	+	-	-	-	-
Memory B cells	+	-	+	+	-	-	+
Plasma blasts	+	+/-	-	+	+	+	+
Plasma cells	-	+/-	-	+	+	+	-

[a] plasma blast และ plasma cells ส่วนหนึ่งไม่มี CD10

[b] classical IgG+ memory B cells ส่วนหนึ่งไม่มี CD27

จนถึงปัจจุบันนี้ก็ยังไม่มีข้อสรุปเกี่ยวกับตารางการฉีดวัคซีนไวรัสตับอักเสบบีที่เหมาะสมที่สุดในผู้ป่วยเอชไอวี ความสำเร็จในการชักนำให้เกิดแอนติบอดีต่อไวรัสตับอักเสบในระดับที่สามารถป้องกันโรคได้และสามารถคงอยู่ได้นานในร่างกายนั้นขึ้นอยู่กับการกระตุ้นภูมิคุ้มกันครั้งแรก (Cruciani et al., 2009; Rey et al., 2000; Van Herck et al., 1998) ผู้ติดเชื้อเอชไอวีที่มีระดับ CD4 T cells สูงจะมีการตอบสนองต่อวัคซีนไวรัสตับอักเสบบีได้ดีกว่า ในทางตรงข้ามผู้ที่มีจำนวนไวรัสสูงจะทำให้การตอบสนองต่อวัคซีนบกพร่อง (Cruciani et al., 2009; Ungulkraiwit et al., 2007) งานวิจัยฉบับนี้เป็นการศึกษาแบบสุ่มเพื่อ

เปรียบเทียบประสิทธิภาพในการกระตุ้นให้เกิดภูมิคุ้มกันต่อไวรัสตับอักเสบบีโดยการเพิ่มความถี่ในการฉีดวัคซีนและการเพิ่มขนาดและความถี่ในการฉีดวัคซีนเปรียบเทียบกับการฉีดวัคซีนไวรัสตับอักเสบบีตามขนาดและความถี่มาตรฐานในผู้ป่วยติดเชื้อเอชไอวีในประเทศไทย ซึ่งคาดว่าจะมีการตอบสนองทางภูมิคุ้มกันที่สูงกว่าการฉีดวัคซีนไวรัสตับอักเสบบีตามขนาดและความถี่มาตรฐาน

7. วิธีการดำเนินการวิจัย (Research methodology)

7.1 แผนงานวิจัยและวิธีการ (Outline of study plan including methodology)

<u>แบบการวิจัย</u> การศึกษาแบบสุ่ม

<u>ประชากรและกลุ่มตัวอย่าง</u> ผู้ป่วยติดเชื้อเอชไอวีที่มารับการรักษาแบบผู้ป่วยนอกที่ห้องตรวจโรคติดเชื้อ ภาควิชาอายุรศาสตร์ คณะแพทยศาสตร์ มหาวิทยาลัยเชียงใหม่ และกลุ่มคนปกติที่ไม่ติดเชื้อเอชไอวี

<u>เกณฑ์การคัดเลือกอาสาสมัครที่ติดเชื้อเอชไอวีเข้าร่วมการศึกษา</u>

1. อายุตั้งแต่ 18 ปีขึ้นไป
2. ได้รับการรักษาด้วยยาต้านไวรัสเอชไอวี
3. CD4 $\geq$ 200 cell/mm3
4. ตรวจเลือดแล้วพบว่าไม่มี HBsAg, Anti-HBs, Anti-HBc และ Anti-HCV
5. ไม่เคยได้รับการฉีดวัคซีนไวรัสตับอักเสบบี
6. ไม่มีการติดเชื้อฉวยโอกาสขณะที่เข้าร่วมการศึกษา
7. ผู้ป่วยที่ยินยอมเข้าร่วมงานวิจัย
8. สามารถมาตามนัดได้

<u>เกณฑ์การคัดเลือกอาสาสมัครกลุ่มคนปกติเข้าร่วมการศึกษา</u>

1. อายุตั้งแต่ 18 ปีขึ้นไป
2. ตรวจเลือดแล้วพบว่าไม่มี HBsAg, Anti-HBs, Anti-HBc และ Anti-HCV และ Anti-HIV
3. ไม่เคยได้รับการฉีดวัคซีนไวรัสตับอักเสบบี
4. ยินยอมเข้าร่วมงานวิจัย
5. สามารถมาตามนัดได้

<u>เกณฑ์การคัดเลือกอาสาสมัครออกจากการศึกษา</u>

1. ตั้งครรภ์หรือให้นมบุตร
2. มีประวัติแพ้ส่วนประกอบของวัคซีน
3. ไม่สามารถมาตามนัดได้

<u>วิธีการวิจัย</u>

คัดกรองอาสาสมัครเป้าหมายตามเกณฑ์การรับอาสาสมัครโดยดำเนินการดังนี้

1. กลุ่มผู้ติดเชื้อเอชไอวี: ติดประกาศและประชาสัมพันธ์แก่ผู้ป่วยที่ห้องตรวจผู้ป่วยโรคติดเชื้อ หรือแพทย์/พยาบาลผู้ดูแลผู้ป่วยเชิญชวนเพื่อรับสมัครผู้ที่ต้องการเข้าร่วมการศึกษา
   กลุ่มคนปกติที่ไม่ติดเชื้อเอชไอวี: ติดประกาศและประชาสัมพันธ์ในบริเวณคณะแพทยศาสตร์ และสถาบันวิจัยวิทยาศาสตร์สุขภาพ
2. อธิบายทำความเข้าใจถึงวัตถุประสงค์และวิธีทำการศึกษาและขอคำให้การยินยอมเข้าร่วมโครงการ
3. ผู้วิจัยเก็บข้อมูลโดยการสัมภาษณ์ในส่วนข้อมูลทั่วไป ได้แก่ เพศ อายุ วันที่ประจำเดือนมาครั้งสุดท้าย วิธีการคุมกำเนิด เก็บข้อมูลจากเวชระเบียนในส่วนของ CD4, Viral load ประวัติการรักษาด้วยยาต้านไวรัส ประวัติการติดเชื้อฉวยโอกาส และตรวจร่างกายตามระบบ เจาะเลือดเพื่อคัดเลือกตามเกณฑ์การรับอาสาสมัครและเกณฑ์การไม่รับอาสาสมัครเข้าร่วมโครงการ <u>ในการเจาะเลือดกลุ่มคนปกติจะทำการให้คำปรึกษาก่อนและหลังการตรวจเชื้อเอชไอวี</u>
4. แบ่งอาสาสมัคร<u>ผู้ติดเชื้อเอชไอวี</u>ออกเป็น 3 กลุ่มโดยใช้วิธีสุ่ม
   กลุ่มที่ 1 กลุ่มขนาดและความถี่มาตรฐาน (กลุ่มควบคุม) อาสาสมัครจะได้รับการฉีดวัคซีนไวรัสตับอักเสบบีขนาด 20 μg เข้ากล้ามที่บริเวณกล้ามเนื้อต้นแขนที่เดือนที่ 0, 1, 6
   กลุ่มที่ 2 กลุ่มขนาดมาตรฐานแต่เพิ่มความถี่ อาสาสมัครจะได้รับการฉีดวัคซีนไวรัสตับอักเสบบีขนาด 20 μg เข้ากล้ามที่บริเวณกล้ามเนื้อต้นแขนที่เดือนที่ 0, 1, 2, 6
   กลุ่มที่ 3 กลุ่มเพิ่มขนาดและความถี่ อาสาสมัครจะได้รับการฉีดวัคซีนไวรัสตับอักเสบบีขนาด 40 μg เข้ากล้ามที่บริเวณกล้ามเนื้อต้นแขนที่เดือนที่ 0, 1, 2, 6
   <u>กลุ่มคนปกติที่ไม่ติดเชื้อเอชไอวี</u>จะได้รับวัคซีนไวรัสตับอักเสบบีที่ขนาดและความถี่มาตรฐาน คือขนาด 20 μg เข้ากล้ามที่บริเวณกล้ามเนื้อต้นแขนที่เดือนที่ 0, 1, 6
5. เจาะเลือดอาสาสมัครทุกคนปริมาณ 23 มิลลิลิตรที่ 1) ก่อนฉีดวัคซีนเข็มแรก 2) ที่ 7 วันหลังการฉีดวัคซีนเข็มแรก 3) ก่อนฉีดวัคซีนแต่ละเข็ม และ 4) ที่ 1 เดือนหลังจากฉีดวัคซีนไวรัสตับอักเสบบีเข็มสุดท้ายเพื่อดูระดับของภูมิคุ้มกันต่อไวรัสตับอักเสบบี (Anti-HBs) (หลอด 6 มล. 1 หลอดเพื่อตรวจหาภูมิคุ้มกันต่อไวรัสตับอักเสบบี หลอด 8.5 มล 2 หลอด เพื่อตรวจหาภูมิคุ้มกันระบบเซลล์)
   ในอาสาสมัครที่พบว่ามีภูมิคุ้มกันต่อไวรัสตับอักเสบบีหลังฉีดวัคซีนเข็มสุดท้ายจะทำการเจาะเลือดอีกครั้งที่ 6 เดือน (ที่ 12 เดือนจากเข็มแรก) เพื่อดูระดับการคงอยู่ของภูมิคุ้มกันต่อไวรัสตับอักเสบบี (Anti-HBs) รวมเป็น 6 ครั้งในกลุ่มควบคุมและ 7 ครั้งในกลุ่มขนาดมาตรฐานเพิ่มความถี่และกลุ่มเพิ่มขนาดและความถี่

หลังจากฉีดวัคซีนไวรัสตับอักเสบบีทุกเข็มจะมีสมุดบันทึกให้อาสาสมัครบันทึกอาการที่ไม่พึงประสงค์จากการฉีดวัคซีน และให้อาสาสมัครนำมาเมื่อมาพบแพทย์ครั้งต่อไป

วัคซีนที่ใช้ในการศึกษา - ใช้วัคซีนไวรัสตับอักเสบที่ผลิตโดยใช้เทคโนโลยีรีคอมบิแนนท์ ดีเอ็นเอ (Recombinant Hepatitis B vaccine) พัฒนาโดยบริษัท Berna Biotech Korea Corporation

6. ในอาสาสมัครที่ตรวจไม่พบภูมิคุ้มกันที่ 1 เดือนหลังจากฉีดวัคซีนไวรัสตับอักเสบบีเข็มสุดท้าย แพทย์ผู้เชี่ยวชาญจะทำการประเมินผลการศึกษาและให้คำแนะนำเป็นกรณีไป
7. การวิเคราะห์ทางห้องปฏิบัติการ

   เลือดที่ได้จะถูกนำมาแยกเป็นสองส่วนคือพลาสม่าและเม็ดเลือด โดยพลาสม่าจะนำไปตรวจดังต่อไปนี้

   - การตรวจเชิงปริมาณหา Anti-HBs Abs โดยใช้เทคนิค ELISA
   - การตรวจเชิงคุณภาพหา HBsAg, Anti-HBc Abs, และ Anti-HCV Abs โดยใช้เทคนิค ELISA
   - การตรวจเชิงคุณภาพหา anti-HIV Abs โดยใช้ rapid test หากให้ผลบวกจะส่งยืนยันผลด้วยวิธี ELISA และ Western blot
   - การตรวจหาจำนวนเม็ดเลือดขาวชนิด CD4 ใช้ชุดน้ำยา BD Tritest three-color reagents
   - การตรวจหา Viral load ใช้ชุดน้ำยา COBAS® AmpliPrep/COBAS® Taqman® HIV-1 Test, version 2.0 สามารถตรวจพบค่า Viral load ต่ำสุดเท่ากับ 20 copies/ml
   - หา affinity ของแอนติบอดีด้วยวิธี ELISA โดยเติมสาร guanidine dissociating หลังจากขั้นตอนของการเติม plasma/serum
   - หาระดับของ interleukin (IL)-6 และ IL-21 ด้วยวิธี ELISA
   - การวิเคราะห์หา HBsAg, Anti-HBs, Anti-HBc, Anti-HCV จะตรวจที่ห้องปฏิบัติภูมิคุ้มกันวิทยา สถาบันวิจัยวิทยาศาสตร์สุขภาพ มหาวิทยาลัยเชียงใหม่ และการตรวจหาจำนวนเม็ดเลือดขาวชนิด CD4, Viral load ตรวจที่ห้องปฏิบัติการภูมิคุ้มกันวิทยา ภาควิชาจุลชีววิทยา คณะแพทยศาสตร์ มหาวิทยาลัยเชียงใหม่
   - การที่มีระดับ Anti-HBs ≥ 10 mIU/mL ถือว่ามีภูมิคุ้มกันต่อไวรัสตับอักเสบบี
   - ส่วนของเม็ดเลือดจะถูกนำไปปั่นแยกเม็ดเลือดขาวชนิดนิวเคลียสเดี่ยว (peripheral blood mononuclear cells: PBMC) ด้วยวิธี gradient centrifugation การหาลักษณะของ B cells จะทำโดยย้อมเซลล์ด้วย monoclonal antibody และตรวจวัดด้วย flow cytometry

- ประเมินคุณสมบัติในการสร้างแอนติบอดีของ circulating memory B cells โดยแยก B cells ด้วยการใช้ magnetic beads ทำการ dilute เซลล์ที่จำนวนเซลล์ต่าง ๆ กัน (limiting dilution) กระตุ้นด้วย polyclonal activator เป็นเวลา 5 วัน หลังจากนั้นเก็บ Supernatant ที่ได้ไปหาปริมาณแอนติบอดีต่อแอนติเจนหรือโปรตีนของชิ้นส่วนต่าง ๆ ของไวรัสตับอักเสบบี ตรวจวัดด้วยวิธี Enzyme-linked immunosorbent assay
- ตรวจวัดภูมิคุ้มกันระบบเซลล์ (cell mediated immunity) โดยหาลักษณะของเซลล์ที่จำเพาะต่อไวรัสตับอักเสบบี ซึ่งทำได้โดยติดฉลาก PBMC ด้วย carboxyfluorescein diacetate succinimidyl ester (CFSE) ซึ่งเป็น fluorescent dye ที่แบ่งไปสู่เซลล์ลูกครึ่งหนึ่งเท่า ๆ กันเมื่อมีการแบ่งตัวของเซลล์ตั้งต้นแต่ละครั้ง CFSE-PBMC ที่แบ่งตัวหลังการกระตุ้นด้วยแอนติเจน 6 วัน จะถูกย้อมทับด้วยแอนติบอดีต่อ surface marker ต่าง ๆ และนำไปวิเคราะห์ด้วย 8-color flow cytometry นอกจากนั้น supernatant จะถูกเก็บไว้ที่ -70 °C เพื่อวิเคราะห์หา cytokines ด้วยวิธี ELISA และ PBMC ส่วนหนึ่งจะถูกกระตุ้นด้วยแอนติเจนเป็นเวลา 16-20 ชั่วโมง ทำการย้อมเซลล์ด้วยแอนติบอดีต่อ cytokines, surface marker และโมเลกุลที่แสดงถึงการเกิด degranulation นำไปวิเคราะห์ด้วย 8-color flow cytometry วิธีนี้จะทำให้สามารถวิเคราะห์จำนวน ลักษณะของเซลล์และคุณสมบัติของเซลล์ได้ในขณะเดียวกัน Transcription factors ที่มีผลต่อการสร้างแอนติบอดีเช่น T-bet, Eomes และ Blimp-1 จะถูกวิเคราะห์ด้วยวิธี Reverse transcription polymerase chain reaction (RT-PCR)

<u>ระยะเวลาศึกษาวิจัย</u>:

เริ่มโครงการหลังจากที่ได้รับอนุมัติจากคณะกรรมการพิจารณาจริยธรรมฯ เรียบร้อยแล้ว และระยะเวลาในการทำวิจัยนาน 3 ปี

7.2 วิธีวิเคราะห์ข้อมูล (Method of data analysis)[1]

โดยข้อมูลทั่วไปของอาสาสมัคร วิเคราะห์ด้วยการแจกแจงความถี่ คำนวณร้อยละหาค่าเฉลี่ยและส่วนเบี่ยงเบนมาตรฐาน

สำหรับข้อมูลที่มีความต่อเนื่องใช้ paired T-test แต่ถ้าเปรียบเทียบระหว่างสองกลุ่มใช้ T-test ส่วนข้อมูลความถี่ใช้ Chi-square test หรือ Fisher exact test ตามความเหมาะสม โดยค่า $p < 0.05$ ถือว่ามีความแตกต่างกันอย่างมีนัยสำคัญทางสถิติ อายุขัยหรือการเปลี่ยนแปลงของแอนติบอดีและ memory B cells จะประเมินโดยการใช้ log-linear mixed-effects regression model

---

[1] วิธีทางสถิติ

การคำนวณขนาดตัวอย่าง

ประมาณการณ์ว่าในกลุ่มที่ได้รับวัคซีนตับอักเสบบีขนาดและความถี่มาตรฐาน (กลุ่มควบคุม) จะมีภูมิคุ้มกันต่อไวรัสตับอักเสบบี (Anti-HBs ≥ 10 mIU/mL) 50% ส่วนในกลุ่มที่ได้รับวัคซีนตับอักเสบบีขนาดมาตรฐานแต่เพิ่มความถี่และกลุ่มที่ได้รับวัคซีนตับอักเสบบีเพิ่มขนาดและความถี่จะมีภูมิคุ้มกันต่อไวรัสตับอักเสบบี (Anti-HBs ≥ 10 mIU/mL) 80% เพื่อที่จะหาความแตกต่างจะต้องมีอาสาสมัครในแต่ละกลุ่มเท่ากับ 41 คน (α = 0.05 and β = 0.20) และคาดการณ์ว่าอัตราของการไม่มาตามนัดจะมีน้อยประมาณ 5% ดังนั้นจะต้องมีอาสาสมัครในแต่ละกลุ่มเท่ากับ 44 คน

7.3 ตารางการดำเนินการวิจัย (Time Table) ระยะเวลา 3 ปี

กิจกรรม	ปีที่ 1				ปีที่ 2				ปีที่ 3			
	1-3	4-6	7-9	10-12	1-3	4-6	7-9	10-12	1-3	4-6	7-9	10-12
1. การจัดซื้อครุภัณฑ์												
2. การผลิตบุคลากรทุนประเภทต่างๆ												
3. การสร้างเครือข่ายวิจัย												
4. การดำเนินโครงการวิจัย(ระบุกิจกรรมต่างๆ)												
4.1 ติดประกาศและประกาศเสียงตามสายเพื่อรับสมัครผู้ที่สนใจเข้าร่วมการศึกษาและคัดเลือกตามเกณฑ์การรับอาสาสมัครและเกณฑ์การไม่รับอาสาสมัครเข้าร่วมโครงการ	↔											
4.2 ฉีดวัคซีน เก็บเลือดตัวอย่าง ติดตามผู้ป่วย	↔	↔	↔				↔	↔	↔			
4.3 เตรียมความพร้อมทางห้องปฏิบัติการ สารเคมี และวัสดุอุปกรณ์ หาสภาวะที่เหมาะสมสำหรับการวิเคราะห์ต่าง ๆ	↔											
4.4 ตรวจวิเคราะห์หาแอนติบอดี และการตอบสนองของภูมิคุ้มกันระบบเซลล์		↔	↔	↔			↔	↔	↔	↔		
4.5 วิเคราะห์ข้อมูล			↔	↔	↔		↔	↔	↔	↔	↔	
4.6 เขียนรายงานฉบับสมบูรณ์และผลงานวิจัยเพื่อตีพิมพ์			↔	↔	↔	↔		↔	↔	↔	↔	↔

8. เอกสารอ้างอิง

(2001). Recommendations for preventing transmission of infections among chronic hemodialysis patients. MMWR Recomm Rep 50, 1-43.

Alper, C.A., Kruskall, M.S., Marcus-Bagley, D., Craven, D.E., Katz, A.J., Brink, S.J., Dienstag, J.L., Awdeh, Z., and Yunis, E.J. (1989). Genetic prediction of nonresponse to hepatitis B vaccine. N Engl J Med 321, 708-712.

Alwayn, I.P., Xu, Y., Basker, M., Wu, C., Buhler, L., Lambrigts, D., Treter, S., Harper, D., Kitamura, H., Vitetta, E.S., *et al.* (2001). Effects of specific anti-B and/or anti-plasma cell immunotherapy on antibody production in baboons: depletion of CD20- and CD22-positive B cells does not result in significantly decreased production of anti-alphaGal antibody. Xenotransplantation 8, 157-171.

Amanna, I.J., Carlson, N.E., and Slifka, M.K. (2007). Duration of humoral immunity to common viral and vaccine antigens. N Engl J Med 357, 1903-1915.

Andre, F.E. (1989). Summary of safety and efficacy data on a yeast-derived hepatitis B vaccine. Am J Med 87, 14S-20S.

Anolik, J.H., Barnard, J., Cappione, A., Pugh-Bernard, A.E., Felgar, R.E., Looney, R.J., and Sanz, I. (2004). Rituximab improves peripheral B cell abnormalities in human systemic lupus erythematosus. Arthritis Rheum 50, 3580-3590.

Averhoff, F., Mahoney, F., Coleman, P., Schatz, G., Hurwitz, E., and Margolis, H. (1998). Immunogenicity of hepatitis B Vaccines. Implications for persons at occupational risk of hepatitis B virus infection. Am J Prev Med 15, 1-8.

Bekker, V., Scherpbier, H., Pajkrt, D., Jurriaans, S., Zaaijer, H., and Kuijpers, T.W. (2006). Persistent humoral immune defect in highly active antiretroviral therapy-treated children with HIV-1 infection: loss of specific antibodies against attenuated vaccine strains and natural viral infection. Pediatrics 118, e315-322.

Bodsworth, N.J., Cooper, D.A., and Donovan, B. (1991). The influence of human immunodeficiency virus type 1 infection on the development of the hepatitis B virus carrier state. J Infect Dis 163, 1138-1140.

Bruguera, M., Cremades, M., Rodicio, J.L., Alcazar, J.M., Oliver, A., Del Rio, G., and Esteban-Mur, R. (1989). Immunogenicity of a yeast-derived hepatitis B vaccine in hemodialysis patients. Am J Med 87, 30S-32S.

Colin, J.F., Cazals-Hatem, D., Loriot, M.A., Martinot-Peignoux, M., Pham, B.N., Auperin, A., Degott, C., Benhamou, J.P., Erlinger, S., Valla, D., and Marcellin, P. (1999). Influence of human

immunodeficiency virus infection on chronic hepatitis B in homosexual men. Hepatology 29, 1306-1310.

N. Pasricha, U. Datta, Y. Chawla, S. Singh, S.K. Arora and A. Sud *et al.*, Immune responses in patients with HIV infection after vaccination with recombinant Hepatitis B virus vaccine, *BMC Infect Dis* **6** (2006), p. 65.

Cornejo-Juarez, P., Volkow-Fernandez, P., Escobedo-Lopez, K., Vilar-Compte, D., Ruiz-Palacios, G., and Soto-Ramirez, L.E. (2006). Randomized controlled trial of Hepatitis B virus vaccine in HIV-1-infected patients comparing two different doses. AIDS Res Ther 3, 9.

M. Das Gracas Sasaki, R. Foccacia and I.J. de Messias-Reason, Efficacy of granulocyte-macrophage colony-stimulating factor (GM-CSF) as a vaccine adjuvant for hepatitis B virus in patients with HIV infection et al, Vaccine 21 (2003), pp. 4545–4549.

Cooper, C.L., Angel, J.B., Seguin, I., Davis, H.L., and Cameron, D.W. (2008). CPG 7909 adjuvant plus hepatitis B virus vaccination in HIV-infected adults achieves long-term seroprotection for up to 5 years. Clin Infect Dis 46, 1310-1314.

Cruciani, M., Mengoli, C., Serpelloni, G., Lanza, A., Gomma, M., Nardi, S., Rimondo, C., Bricolo, F., Consolaro, S., Trevisan, M., and Bosco, O. (2009). Serologic response to hepatitis B vaccine with high dose and increasing number of injections in HIV infected adult patients. Vaccine 27, 17-22.

da Mota Silveira Sasaki, M.G., Sobroza De Mello, R., Focaccia Siciliano, R., and Wang, L. (1998). Response of HIV/AIDS Patients to Hepatitis B Recombinant Vaccine. Braz J Infect Dis 2, 236-240.

Denis, F., Adjide, C.C., Rogez, S., Delpeyroux, C., Rogez, J.P., and Weinbreck, P. (1997). [Seroprevalence of HBV, HCV and HDV hepatitis markers in 500 patients infected with the human immunodeficiency virus]. Pathol Biol (Paris) 45, 701-708.

DiLillo, D.J., Hamaguchi, Y., Ueda, Y., Yang, K., Uchida, J., Haas, K.M., Kelsoe, G., and Tedder, T.F. (2008). Maintenance of long-lived plasma cells and serological memory despite mature and memory B cell depletion during CD20 immunotherapy in mice. J Immunol 180, 361-371.

Fonseca, M.O., Pang, L.W., de Paula Cavalheiro, N., Barone, A.A., and Heloisa Lopes, M. (2005). Randomized trial of recombinant hepatitis B vaccine in HIV-infected adult patients comparing a standard dose to a double dose. Vaccine 23, 2902-2908.

Francisci, D., Baldelli, F., Papili, R., Stagni, G., and Pauluzzi, S. (1995). Prevalence of HBV, HDV and HCV hepatitis markers in HIV-positive patients. Eur J Epidemiol 11, 123-126.

Gilson, R.J., Hawkins, A.E., Beecham, M.R., Ross, E., Waite, J., Briggs, M., McNally, T., Kelly, G.E., Tedder, R.S., and Weller, I.V. (1997). Interactions between HIV and hepatitis B virus in homosexual men: effects on the natural history of infection. AIDS 11, 597-606.

Hadler, S.C., Judson, F.N., O'Malley, P.M., Altman, N.L., Penley, K., Buchbinder, S., Schable, C.A., Coleman, P.J., Ostrow, D.N., and Francis, D.P. (1991). Outcome of hepatitis B virus infection in homosexual men and its relation to prior human immunodeficiency virus infection. J Infect Dis 163, 454-459.

Horvath, J., and Raffanti, S.P. (1994). Clinical aspects of the interactions between human immunodeficiency virus and the hepatotropic viruses. Clin Infect Dis 18, 339-347.

Iwakoshi, N.N., Lee, A.H., and Glimcher, L.H. (2003). The X-box binding protein-1 transcription factor is required for plasma cell differentiation and the unfolded protein response. Immunol Rev 194, 29-38.

Kalinowska-Nowak, A., Bociaga-Jasik, M., Garlicki, A., and Mach, T. (2007). [Efficacy of vaccination against hepatitis B in adult with HIV infection]. Przegl Epidemiol 61, 339-347.

Kaplan, J.E., Benson, C., Holmes, K.H., Brooks, J.T., Pau, A., Masur, H., Centers for Disease, C., Prevention, National Institutes of, H., and America, H.I.V.M.A.o.t.I.D.S.o. (2009). Guidelines for prevention and treatment of opportunistic infections in HIV-infected adults and adolescents: recommendations from CDC, the National Institutes of Health, and the HIV Medicine Association of the Infectious Diseases Society of America. MMWR Recomm Rep 58, 1-207; quiz CE201-204.

Krogsgaard, K., Lindhardt, B.O., Nielson, J.O., Andersson, P., Kryger, P., Aldershvile, J., Gerstoft, J., and Pedersen, C. (1987). The influence of HTLV-III infection on the natural history of hepatitis B virus infection in male homosexual HBsAg carriers. Hepatology 7, 37-41.

Lewden, C., Salmon, D., Morlat, P., Bevilacqua, S., Jougla, E., Bonnet, F., Heripret, L., Costagliola, D., May, T., Chene, G., and Mortality study, g. (2005). Causes of death among human immunodeficiency virus (HIV)-infected adults in the era of potent antiretroviral therapy: emerging role of hepatitis and cancers, persistent role of AIDS. Int J Epidemiol 34, 121-130.

Loke, R.H., Murray-Lyon, I.M., Coleman, J.C., Evans, B.A., and Zuckerman, A.J. (1990). Diminished response to recombinant hepatitis B vaccine in homosexual men with HIV antibody: an indicator of poor prognosis. J Med Virol 31, 109-111.

Mandelli, C., Cesana, M., Ferroni, P., Lorini, G.P., Aimo, G.P., Tagger, A., Bianchi, P.A., and Conte, D. (1988). HBV, HDV and HIV infections in 242 drug addicts: two-year follow-up. Eur J Epidemiol 4, 318-321.

Manz, R.A., Hauser, A.E., Hiepe, F., and Radbruch, A. (2005). Maintenance of serum antibody levels. Annu Rev Immunol 23, 367-386.

Mendes-Correa, M.C., Barone, A.A., Cavalheiro, N., Tengan, F.M., and Guastini, C. (2000). Prevalence of hepatitis B and C in the sera of patients with HIV infection in Sao Paulo, Brazil. Rev Inst Med Trop Sao Paulo 42, 81-85.

Ministério da Saúde do Brasil, F.N.d.S. (2002). Recomendações para vacinação em pessoas infectadas pelo HIV. FUNASA *Novembro.*

Moir, S., Ho, J., Malaspina, A., Wang, W., DiPoto, A.C., O'Shea, M.A., Roby, G., Kottilil, S., Arthos, J., Proschan, M.A., *et al.* (2008). Evidence for HIV-associated B cell exhaustion in a dysfunctional memory B cell compartment in HIV-infected viremic individuals. J Exp Med 205, 1797-1805.

Moser, K., Tokoyoda, K., Radbruch, A., MacLennan, I., and Manz, R.A. (2006). Stromal niches, plasma cell differentiation and survival. Curr Opin Immunol 18, 265-270.

Muehlinghaus, G., Cigliano, L., Huehn, S., Peddinghaus, A., Leyendeckers, H., Hauser, A.E., Hiepe, F., Radbruch, A., Arce, S., and Manz, R.A. (2005). Regulation of CXCR3 and CXCR4 expression during terminal differentiation of memory B cells into plasma cells. Blood 105, 3965-3971.

Ockenga, J., Tillmann, H.L., Trautwein, C., Stoll, M., Manns, M.P., and Schmidt, R.E. (1997). Hepatitis B and C in HIV-infected patients. Prevalence and prognostic value. J Hepatol 27, 18-24.

Okada, T., Miller, M.J., Parker, I., Krummel, M.F., Neighbors, M., Hartley, S.B., O'Garra, A., Cahalan, M.D., and Cyster, J.G. (2005). Antigen-engaged B cells undergo chemotaxis toward the T zone and form motile conjugates with helper T cells. PLoS Biol 3, e150.

Pasricha, N., Datta, U., Chawla, Y., Singh, S., Arora, S.K., Sud, A., Minz, R.W., Saikia, B., Singh, H., James, I., and Sehgal, S. (2006). Immune responses in patients with HIV infection after vaccination with recombinant Hepatitis B virus vaccine. BMC Infect Dis 6, 65.

Rey, D., Krantz, V., Partisani, M., Schmitt, M.P., Meyer, P., Libbrecht, E., Wendling, M.J., Vetter, D., Nicolle, M., Kempf-Durepaire, G., and Lang, J.M. (2000). Increasing the number of hepatitis B vaccine injections augments anti-HBs response rate in HIV-infected patients. Effects on HIV-1 viral load. Vaccine 18, 1161-1165.

Salmon-Ceron, D., Lewden, C., Morlat, P., Bevilacqua, S., Jougla, E., Bonnet, F., Heripret, L., Costagliola, D., May, T., Chene, G., and Mortality study, g. (2005). Liver disease as a major cause of death among HIV infected patients: role of hepatitis C and B viruses and alcohol. J Hepatol 42, 799-805.

Sasaki, M.G., Foccacia, R., and de Messias-Reason, I.J. (2003). Efficacy of granulocyte-macrophage colony-stimulating factor (GM-CSF) as a vaccine adjuvant for hepatitis B virus in patients with HIV infection. Vaccine 21, 4545-4549.

Scharschmidt, B.F., Held, M.J., Hollander, H.H., Read, A.E., Lavine, J.E., Veereman, G., McGuire, R.F., and Thaler, M.M. (1992). Hepatitis B in patients with HIV infection: relationship to AIDS and patient survival. Ann Intern Med 117, 837-838.

Shaw, F.E., Jr., Guess, H.A., Roets, J.M., Mohr, F.E., Coleman, P.J., Mandel, E.J., Roehm, R.R., Jr., Talley, W.S., and Hadler, S.C. (1989). Effect of anatomic injection site, age and smoking on the immune response to hepatitis B vaccination. Vaccine 7, 425-430.

Sinicco, A., Raiteri, R., Sciandra, M., Bertone, C., Lingua, A., Salassa, B., and Gioannini, P. (1997). Coinfection and superinfection of hepatitis B virus in patients infected with human immunodeficiency virus: no evidence of faster progression to AIDS. Scand J Infect Dis 29, 111-115.

Soriano, V., Puoti, M., Bonacini, M., Brook, G., Cargnel, A., Rockstroh, J., Thio, C., and Benhamou, Y. (2005). Care of patients with chronic hepatitis B and HIV co-infection: recommendations from an HIV-HBV International Panel. AIDS 19, 221-240.

Tarlinton, D. (2006). B-cell memory: are subsets necessary? Nat Rev Immunol 6, 785-790.

Tokoyoda, K., Egawa, T., Sugiyama, T., Choi, B.I., and Nagasawa, T. (2004). Cellular niches controlling B lymphocyte behavior within bone marrow during development. Immunity 20, 707-718.

Treitinger, A., Spada, C., Silva, E.L., Miranda, A.F., Oliveira, O.V., Silveira, M.V., Verdi, J.C., and Abdalla, D.S. (1999). Prevalence of Serologic Markers of HBV and HCV Infection in HIV-1 Seropositive Patients in Florianopolis, Brazil. Braz J Infect Dis 3, 1-5.

Ungulkraiwit, P., Jongjirawisan, Y., Atamasirikul, K., and Sungkanuparph, S. (2007). Factors for predicting successful immune response to hepatitis B vaccination in HIV-1 infected patients. Southeast Asian J Trop Med Public Health 38, 680-685.

Vallerskog, T., Gunnarsson, I., Widhe, M., Risselada, A., Klareskog, L., van Vollenhoven, R., Malmstrom, V., and Trollmo, C. (2007). Treatment with rituximab affects both the cellular and the humoral arm of the immune system in patients with SLE. Clin Immunol 122, 62-74.

Van Herck, K., Van Damme, P., Thoelen, S., and Meheus, A. (1998). Long-term persistence of anti-HBs after vaccination with a recombinant DNA yeast-derived hepatitis B vaccine: 8-year results. Vaccine 16, 1933-1935.

Weber, D.J., Rutala, W.A., Samsa, G.P., Santimaw, J.E., and Lemon, S.M. (1985). Obesity as a predictor of poor antibody response to hepatitis B plasma vaccine. JAMA 254, 3187-3189.

Wood, R.C., MacDonald, K.L., White, K.E., Hedberg, C.W., Hanson, M., and Osterholm, M.T. (1993). Risk factors for lack of detectable antibody following hepatitis B vaccination of Minnesota health care workers. JAMA 270, 2935-2939.

Zajac, B.A., West, D.J., McAleer, W.J., and Scolnick, E.M. (1986). Overview of clinical studies with hepatitis B vaccine made by recombinant DNA. J Infect 13 *Suppl A*, 39-45.

9. ประโยชน์ที่จะได้รับ

1. เป็นแนวทางในการดูแลผู้ป่วยเอชไอวีที่ยังไม่มีภูมิคุ้มกันต่อไวรัสตับอักเสบบี
2. เป็นข้อมูลพื้นฐานในการปรับเปลี่ยนแนวปฏิบัติในการฉีดวัคซีนตับอักเสบบีในผู้ป่วยติดเชื้อเอชไอวีในประเทศไทย
3. เข้าใจกลไกการตอบสนองของภูมิคุ้มกันทั้งระบบสารน้ำและระบบเซลล์ ต่อวัคซีนไวรัสตับอักเสบบี ทั้งในคนปกติและผู้ติดเชื้อเอชไอวีที่ได้รับวัคซีนขนาดต่าง ๆ

หน้างบประมาณ

10. รายละเอียดงบประมาณที่เสนอขอ (Proposed budget) รวมทั้งสิ้น 3,444,000 บาท (ตัวอักษร สามล้านสี่แสนสี่หมื่นสี่พันบาท) [งบประมาณในตารางแสดงหน่วยล้านบาท]

กิจกรรม	ปีที่ 1
1. การจัดซื้อครุภัณฑ์	-
2. ทุนสร้างนักวิจัยประเภทต่าง ๆ	-
3. ทุนสร้างเครือข่ายวิจัย (ค่าใช้จ่ายต่อทุน)	-
4. งบประมาณ	
4.1 หมวดค่าจ้างลูกจ้างชั่วคราว	
- นักเทคนิคการแพทย์เต็มเวลา คุณวุฒิปริญญาตรี ในอัตรา 12,000 บาท/เดือน จำนวน 1 คน	144,000
4.2 หมวดค่าตอบแทน	
- หัวหน้าโครงการ (เงินเดือน 10,000 บาท)	120,000
- นักวิจัยในโครงการวิจัยย่อย 2 คน (เงินเดือน 5,000 บาท/คน)	120,000
4.3 หมวดค่าใช้สอย	
- ค่าตอบแทนอาสาสมัครคนละ 300 บาท ต่อการมาตามนัด 1 ครั้ง	286,000
- ค่าใช้สอยอื่น ๆ เช่น ค่าโทรศัพท์เพื่อติดต่อประสานงาน และติดตามอาการอาสาสมัคร	12,000
4.4 หมวดค่าวัสดุ	
- ค่าวัคซีนไวรัสตับอักเสบบี 616 เข็ม ๆ ละ 300 บาท	186,000
- ค่าแยกจัดเก็บพลาสม่าและเม็ดเลือดขาว 1,144 ตัวอย่าง ๆ ละ 400 บาท	372,000
- ค่าตรวจวิเคราะห์ HBsAg	30,000
- ค่าตรวจวิเคราะห์ Anti- HBs Ab	160,000
- ค่าตรวจวิเคราะห์ Anti- HBc Ab	45,000
- ค่าตรวจวิเคราะห์ Anti- HCV Ab	55,000
- ค่าตรวจวิเคราะห์ Anti- HIV Ab	18,000
- ค่าอุปกรณ์เก็บเลือด เช่นเข็มเจาะเลือด หลอดเก็บเลือด	190,000
- สารเคมี media, buffer, sheath fluid, fetal calf serum	300,000
- Monoclonal antibodies	800,000
- วัสดุห้องปฏิบัติการ เช่น tissue culture plates, centrifuge tubes, pipette tips, disposable serological pipettes, bottle top filter, transfer pipettes, polystyrene tubes, ELISA plate เป็นต้น	350,000
- ค่าวัสดุสำนักงาน วัสดุโฆษณาและเผยแพร่	16,000
4.5 หมวดค่าสาธารณูปโภค	240,000
**รวม**	***3,444,000***

ลงนามผู้วิจัย [illegible] วันที่ 15 [illegible] 53 ....

โดยได้งบประมาณมาจากงบมหาวิทยาลัยวิจัย ซึ่งจะพิจารณาอนุมัติงบประมาณเป็นรายปี โดยจะขอยกเว้นค่าธรรมเนียมพิจารณาโครงการวิจัย (Submission fee) และค่าบริหารโครงการวิจัย (overhead charge) เนื่องจากเป็นงบประมาณแผ่นดิน

ขอรับรองว่า ข้อความที่กรอกในแบบฟอร์มนี้เป็นความจริงและสอดคล้องกับข้อเสนอโครงการวิจัยฉบับสมบูรณ์ และผู้กรอกข้อความเข้าใจความหมายโดยชัดเจนทุกประการ พร้อมกันนี้ได้แนบข้อเสนอโครงการวิจัยฉบับสมบูรณ์และเอกสารอื่นๆ ที่เกี่ยวข้องแล้ว

ลงชื่อ..

(แพทย์หญิง กนกพร ไชยกลาง)

ผู้ดำเนินงานวิจัย

ลงชื่อ..

(ดร.จิรประภา วิภายา)

นักวิจัย

ลงชื่อ..

(ศาสตราจารย์นายแพทย์ขวัญชัย ศุภรัตน์ภิญโญ)

อาจารย์ที่ปรึกษา

ลงชื่อ..

(ผู้ช่วยศาสตราจารย์แพทย์หญิงรมณีย์ ชัยวาฤทธิ์)

ผู้ร่วมวิจัย

ลงชื่อ..

(อาจารย์นายแพทย์นนทกานต์ นันทจิต)

ผู้ร่วมวิจัย

ความเห็นผู้บังคับบัญชาชั้นต้น

เห็นสมควรอนุมัติ

(รองศาสตราจารย์นายแพทย์วีระศักดิ์ นาวารวงศ์)

หัวหน้าภาควิชาอายุรศาสตร์
