# Supplementary material for: Comparison of Immunogenicity and Safety of Four Doses and Four Double Doses vs. Standard Doses of Hepatitis B Vaccination in HIV-Infected Adults: A Randomized, Controlled Trial
Source: PLoS One. 2013 Nov 12;8(11):e80409. doi: 10.1371/journal.pone.0080409 (PMC3827227; doi:10.1371/journal.pone.0080409)
Supplement: Protocol S2 — English Translation of Trial Protocol. (DOCX) [file pone.0080409.s003.docx]

**(Stamped submission date: 14 December 2010)**

**Study Protocol (brief translation)**

**1. Title**: Efficacy of modified recombinant hepatitis B vaccination schedule in HIV-Infected adult subjects: a randomized control trial

**2. Department:** Infectious Disease

**3. Investigators:** Kanokporn Chaiklang, MD, Jiraprapa Wipasa, Ph.D, Khuanchai Supparatpinyo, MD, Romanee Chaiwarith, MD, MHS, Nontakan Nuntachit, MD

**4. Research objectives:**

4.1 Primary objectives: to evaluate the efficacy of the HBV vaccination regimens using either increasing frequency or increasing dose and frequency compared with the current standard regimen in HIV-infected adults

4.2 Secondary objectives: to evaluate

- phenotypic features of B-cells after HBV vaccination

- levels of IL-6, Il-10, and IL-21 after HBV vaccination

- antibody levels and properties of memory B-cells after HBV vaccination

- cell-mediated immunity after HBV vaccination

- persistence of antibody response after HBV vaccination

**5. Background**:

**6. Rationale:**

**7. Research methodology**

**7.1 Outline of study plan**

**Study design:** A prospective randomized control trial

**Study population**: HIV-infected individuals attending HIV Clinic at Chiang Mai University Hospital, and healthy controls

**Inclusion criteria:**

1. Age ≥ 18 years old
2. Treated with antiretroviral drugs
3. CD4+ cell count >=200 cells/mm^3^
4. Negative for hepatitis B surface antigen (HBsAg), antibody to hepatitis B surface antigen (anti-HBs), and antibody to hepatitis B core antigen (anti-HBc)
5. No history of previous HBV vaccination
6. No active opportunistic infections (at the time of screening)
7. Compiled to the follow-up schedule

**Exclusion criteria:**

1. Pregnancy or lactation
2. History of hypersensitivity to any component of the vaccine
3. Loss to follow-up

**Study plans:**

1. Recruitment advertisement at the Faculty of Medicine and Research Institute for Health Sciences
2. Informed consent process
3. Data collection, pre- and post-test counselling, and follow-up schedule
4. Block randomization: participants will be randomly assigned to one of 3 parallel arms

1) Standard doses group: receiving 3 intramuscular injections of 20 μg of recombinant HBV vaccine (Hepavax-Gene^®^ Berna, Korea) at months 0, 1, and 6

2) 4 doses group: receiving 4 intramuscular doses of 20 μg of the same vaccine at months 0, 1, 2, and 6

3) 4 double doses group: receiving 4 intramuscular double doses (40 μg) at months 0, 1, 2, and 6

1. Specimen collections
2. Recommendation for non-responders
3. Laboratory evaluation

**Duration of study**: 3 years after study approval

**7.2 Data analysis**

Demographic data: frequency, percentage, mean, SD

Continuous data: paired T-test, frequency: chi-square or Fisher exact tests, significance level: 0.05, changes of antibody levels and memory B-cells: log-linear mixed-effects regression model

Sample size calculation: Standard group response=50%, 4-doses and 4-double doses groups response= 80%, α=0.05, β=0.20, loss to follow-up rate=5%, n=44/group

**7.3 Time table**

**8. References**
